# The Effects of Calcium Channel Blockers in the Prevention of Stroke in Adults with Hypertension: A Meta-Analysis of Data from 273,543 Participants in 31 Randomized Controlled Trials

**DOI:** 10.1371/journal.pone.0057854

**Published:** 2013-03-06

**Authors:** Gui Jv Chen, Mao Sheng Yang

**Affiliations:** Laboratory of Disorder Genes and Department of Pharmacology, College of Pharmacy, Chongqing Medical University, Chongqing, People’s Republic of China; University of British Columbia, Canada

## Abstract

**Background:**

Hypertension is a major risk factor for the development of stroke. It is well known that lowering blood pressure decreases the risk of stroke in people with moderate to severe hypertension. However, the specific effects of calcium channel blockers (CCBs) against stroke in patients with hypertension as compared to no treatment and other antihypertensive drug classes are not known.

**Methods and Findings:**

This systematic review and meta-analysis of randomized controlled trials (RCTs) evaluated CCBs effect on stroke in patients with hypertension in studies of CCBs versus placebo, angiotensin-converting-enzyme inhibitors (ACEIs), β-adrenergic blockers, and diuretics. The PUBMED, MEDLINE, EMBASE, OVID, CNKI, MEDCH, and WANFANG databases were searched for trials published in English or Chinese during the period January 1, 1996 to July 31, 2012. A total of 177 reports were collected, among them 31 RCTs with 273,543 participants (including 130,466 experimental subjects and 143,077 controls) met the inclusion criteria. In these trials a total of 9,550 stroke events (4,145 in experimental group and 5,405 in control group) were reported. CCBs significantly decreased the incidence of stroke compared with placebo (OR = 0.68, 95% CI 0.61–0.75, p<1×10^−5^), β-adrenergic blockers combined with diuretics (OR = 0.89, 95% CI 0.83–0.95, p = 7×10^−5^) and β-adrenergic blockers (OR = 0.79, 95% CI 0.72–0.87, p<1×10^−5^), statistically significant difference was not found between CCBs and ACEIs (OR = 0.92, 95% CI 0.8–1.02, p = 0.12) or diuretics (OR = 0.95, 95% CI 0.84–1.07, p = 0.39).

**Conclusion:**

In a pooled analysis of data of 31 RCTs measuring the effect of CCBs on stroke, CCBs reduced stroke more than placebo and β-adrenergic blockers, but were not different than ACEIs and diuretics. More head to head RCTs are warranted.

## Introduction

Hypertension (MIM #14500) is one of the most common chronic diseases, and the most frequent reason for people to consult their doctor and take medication. Hypertension can burst a blood vessel or/and accelerate narrowing of arteries in the brain to cause a stroke which, if not lethal, can result in many catastrophic complications such as paralysis, aphasia, coma and so forth. The damage to the brain cannot be repaired, so the only rational approach is prevention. Hypertension is a major risk factor for the development of stroke. In 1964, it was first demonstrated that antihypertensive agents could reduce the risk of strokes [1]. It is well known that lowering blood pressure decreases the risk of stroke in people with moderate to severe hypertension [2]. There are eight classifications of antihypertensive agents in use today: α-adrenergic blockers, angiotensin-converting enzyme inhibitors (ACEIs), angiotensin II receptor antagonists, antiadrenergic agents, β-adrenergic blockers or β blockers, calcium channel blockers (CCBs), diuretics, and vasodilators. Despite the widespread use of blood-pressure-lowering agents which one is better against the development of stroke is unclear [3]. Controlling blood pressure in the patients with hypertension or/and stroke has important clinical implications including improved prognosis, reduced mortality and so on [4]. Angiotensin-converting enzyme inhibitors, β-adrenergic blockers, calcium channel blockers and diuretics are used extensively and listed as the first-line agents in the 1989 WHO/International Society of Hypertension Guidelines [5]. Because each study may have insufficient power to detect the effect of calcium channel blockers against stroke in the patients with hypertension; a meta-study to accumulate data from different studies may address this issue, and the specific effects of CCBs against stroke in patients with hypertension as compared to no treatment and other antihypertensive drug classes are not known. Therefore, the major goal of this work was to perform a systematic review and a meta-analysis of the published data and to figure out whether calcium-channel blockers are better than other first-line antihypertensive agents in the prevention of stroke, as well as to quantify the potential heterogeneity between different studies.

## Methods

### Data Sources

The PUBMED, MEDLINE, EMBASE, OVID, CNKI, MEDCH, and WANFANG databases were searched for trials published in English or Chinese during the period January 1, 1996 to July 31, 2012. In addition, all references cited in these studies and previously published review articles were reviewed to identify additional works not indexed by the above databases. Search terms were “antihypertensive agents”, “placebo”, “hypertension”, “diuretics”, “beta-blockers” or “β-adrenergic blockers”, “angiotensin-converting-enzyme inhibitors”, “calcium channel blockers”, “vasodilator agents”, and “stroke”. Bibliographies of studies were also reviewed.

### Study Selection

**Figure 1:**
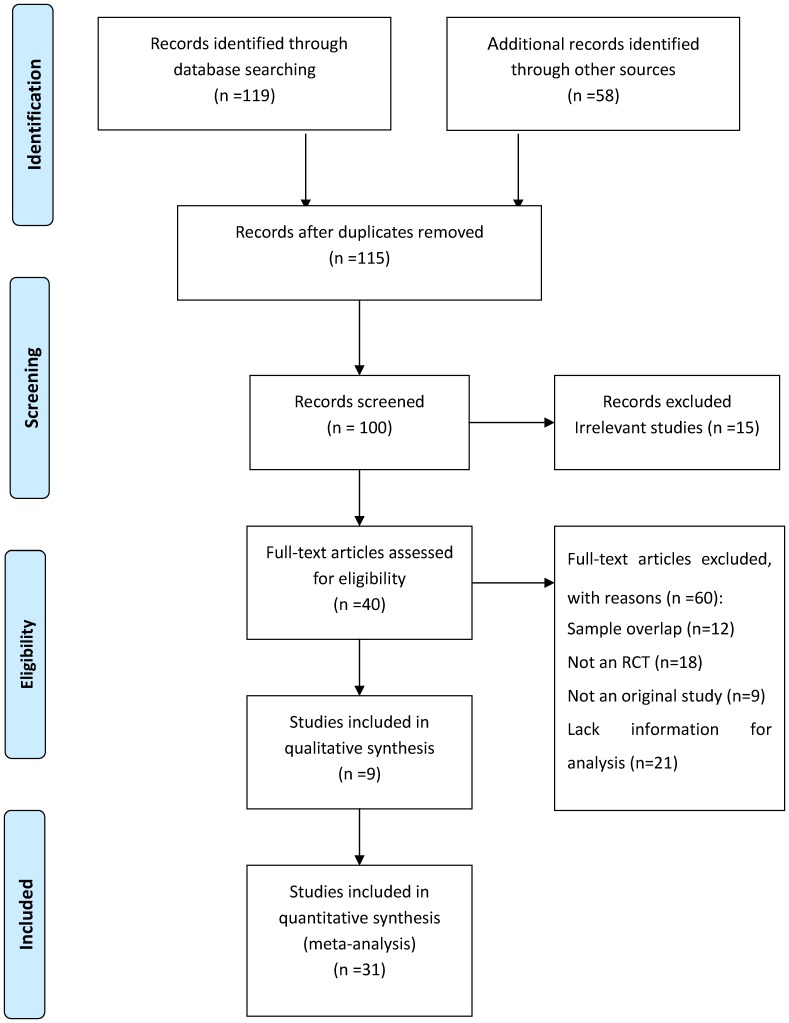
A schematic diagram for the search strategy of published reports.

A total of 177 published studies were identified using the screening procedure shown in Figure 1 (see Supplementary Information online). Among them, fifty-eight are systematic reviews and meta-analyses, one hundred and nineteen are randomized controlled trials. After searching, the following information was extracted: author, ethnicity of research subjects, year of publication, numbers of hypertension- and stroke-cases, medicine of treatment, age of patients, and years of followed-up. Studies were eligible for inclusion if they were randomized controlled trials and reported on stroke risk associated with the current use of the first-line antihypertensive agents in population settings.

### Quality Assessment

Eligible studies must meet the following inclusion criteria: (1) with original data being independent among studies if more than one studies have overlapping subjects, only the study with bigger/biggest sample size was selected; (2) with the numbers of hypertension- and stroke-cases clearly provided; (3) with data of the first-line antihypertensive agents and/or placebo; (4) with the research design of randomized controlled trials, which is the best approach to evaluating new treatments, to challenging the efficacy of the old one, and to comparing the efficacy of different treatments [6]–[7]. All the available information was independently extracted by two investigators and no inconsistency was discovered. The quality assessment of evidence and an overall risk of bias assessment for each included study were evaluated by GRADEprofiler software version 3.2.2 and RevMan version 5.0 (see Supplementary Information online), respectively.

### Statistical Analysis

Publication bias was detected by Egger’s linear regression test, which measures funnel plot asymmetry on the scale of odds ratio (OR) [8]. Heterogeneity between studies was tested by Cochran’s Q-statistic test [9] and *I* ^2^ = 100% × (Q-df) ÷ Q [10], respectively. The *I* ^2^ metric is independent of the number of studies in the meta-analysis, and ranges between 0 and 100% (*I* ^2^<25%: no heterogeneity; *I* ^2^ = 25%–50%: moderate heterogeneity; *I* ^2^ = 50%–75%: high heterogeneity; *I* ^2^>75%: extreme heterogeneity). Heterogeneity was considered statistically significant when *p*<0.05 [11]. If results were heterogeneous, the random effects model was used for meta-analysis. OR was pooled using the method of DerSimonian and Laird, and 95% confidence interval (CI) was constructed using Woolf’s method. The statistical analysis was conducted by the statistical package RevMan version 5.0 (The Cochrane collaboration, Oxford, England). A *p* value of less than 0.05 was considered as statistical significant.

## Results

**Figure 2:**
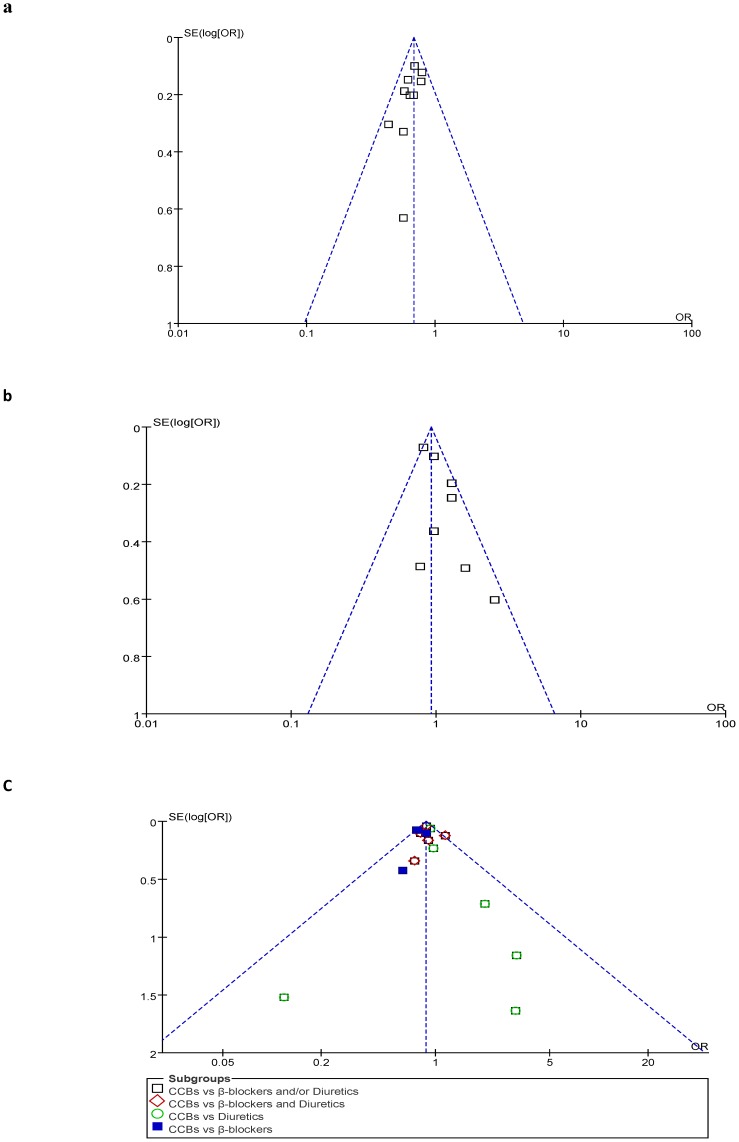
Funnel plots of odds ratios for all studies in the meta-analyses. (**a**) Calcium channel blockers vs Placebo, (**b**) Calcium channel blockers vs ACEIs, and (**c**) Calcium channel blockers vs Diuretics or/and β blockers. No evidence of publication bias was found in any of them.

The derivation of the databases and published articles is described in Figure 1. A total of one hundred and seventy-seven studies concerning the stroke risk associated with the use of antihypertensive agents in the patients with hypertension were identified. Among them, one hundred and forty-six studies were excluded for (1) No numbers of hypertension- and stroke-cases; (2) Data duplication; (3) Not written in English or Chinese; (4) No randomized controlled trials; (5) Data missing or lacking; (6) No control group. Therefore, thirty-one studies [3]–[5], [12]–[39] and a total of 273,543 participants (including 130,466 experimental subjects and 143,077 controls) matched the inclusion criteria and were selected for the statistical test; and a total of 9,550 stroke events (4,145 in experimental group and 5,405 in control group) were reported (see Table 1). The quantity and quality of original investigations play an important role in determining the quality of the meta-analysis. To controlling the publication bias, the funnel test was performed (see Figure 2). No evidence of publication bias was found for the included thirty-one studies. Our analysis also indicated that the heterogeneity between studies was not statistical significance (*p*>0.05), therefore, the fixed effects model was used for the meta-analysis. The results of quality assessment for each included study indicated that among the included thirty-one studies, twenty-eight reports [3]–[5], [12]–[15], [17]–[25], [27]–[32], [34]–[39] were high quality and the remaining three studies [16], [26], [33] were moderate quality (see Table 1 and Supplementary Information online). The overall quality of the evidence was high in our statistical tests.

**Table 1:** Characteristics of 31 randomized controlled trials included in the meta-analyses.

Source year [reference]	Ethnicity	Treatment	Cases of Hypertension		Age of Cases (Mean±SD)		Years of followed up	Cases of Stroke		Incidence of Stroke (%)		Quality of the evidence	Overall risk of bias assessment
			Experimental (Male %)	Control (Male %)	Experimental	Control		Experimental	Control	Experimental	Control	(GRADE)	(RevMan)
**CCBs vs Placebo**													
Poole-Wilson PA et al 2004 [16]	Europe	Nifedipine vs Placebo	3825(80)	3840(79)	63.5±9.3	63.4±9.3	≥4.9	77	99	2	2.6	Moderate	High
Lubsen J et al 2005 [14]	Europe	Nifedipine vs Placebo	1795	2002	61.8±9.4	65.0±8.9	≥5.5	123	171	6.9	8.5	High	Low
Turnbull F 2003 [23]	Europe	CCBs vs Placebo	3794	3688	65	65	≥4	76	119	2	3.2	High	Unclear
Liu L et al 2005 [21]	Asia	Felodipine vs Placebo	4841(61.8)	4870(60.5)	61.5±7.1	61.5±7.2	≥3.5	177	251	3.7	5.2	High	Low
Berl T et al 2003 [37]	Europe	Amlodipine vs Placebo	567(63)	569(71)	59.1±7.9	58.3±8.2	≥2.6	15	26	2.6	4.6	High	Low
Tuomilehto J et al 1999 [38]	Europe	Nitrendipine vs Placebo	2146	2057	≥60	≥60	≥2	42	62	2	3	High	Unclear
Dens JA et al 2001 [25]	Europe	Nisoldipine vs Placebo	408(82)	411(79)	60±9	60±9	≥3	4	7	1	1.7	High	Unclear
Gong L et al 1996 [26]	Asia	Nifedipine vs Placebo	817	815	66.2±5.1	66.7±5.4	≥2.5	16	36	2	4.4	Moderate	High
Liu L et al 1998 [15]	Asia	Nitrendipine vs Placebo	1253(65.0)	1141(63.6)	66.7±5.7	66.4±5.4	≥4	45	59	3.6	5.2	High	Unclear
Staessen JA et al 1998 [12]	Europe	Nitrendipine vs Placebo	2398(32.5)	2297(33.8)	70.2±6.7	70.3±6.7	≥2	47	77	2	3.4	High	Unclear
Total			21844	21690				622	907	2.8	4.2		
**CCBs vs ACEIs**													
Estacio RO et al 1998 [35]	Europe	Nisoldipine vs Enalapril	235(68.1)	235(66.8)	57.2±8.2	57.7±8.4	≥5	11	7	4.7	3	High	Unclear
Leenen FH et al 2005 [20]	Europe	Amlodipine vs Lisinopril	9048(52.7)	9054(53.8)	66.8±7.8	66.8±7.8	≥4	377	457	4.2	5	High	Low
Fukui T et al 2003 [36]	Asia	Amlodipine vs Candesartan	2349	2354	20–85	20–85	≥3.2	60	47	2.6	2	High	Unclear
Song Y et al 2011 [4]	Asia	Levamlodipine Beaylate vs Enapril	69(52.2)	68(51.5)	63.32±6.15	61.85±5.19	≥1	9	11	13	16.2	High	Unclear
Tatti P et al 1998 [13]	Europe	Amlodipine vs Fosinopril	191(55.5)	189(63.5)	62.8±0.5	63.3±0.4	≥3.5	10	4	5.2	2.1	High	Unclear
Hansson L et al 1999 [29]	Europe	CCBs vs ACEIs	2196(34.0)	2205(33.7)	75.9	76.1	≥5	207	215	1	9.8	High	Low
Schrader J et al 2005 [39]	Europe	Nitrendipine vs Eprosartan	671 (54.8)	681(53.6)	67.7±10.4	68.1±9.5	≥2.5	39	31	5.8	4.6	High	Low
Ekbom T et al 2004 [18]	Europe	CCBs vs ACEIs	752(26.6)	772(26.8)	76.5	76.6	≥5	15	16	2	2.1	High	Unclear
Total			15511	15558				728	788	4.7	5.1		
**CCBs vs β** **blockers or/and Diuretics**													
ALLHAT 2002 [27]	Europe	Amlodipine vs Chlorthalidone	9048(52.7)	15255(53.0)	66.9±7.7	66.9±7.7	≥4.9	377	675	4.2	4.4	High	Low
Rothwell PM et al 2010 [17]	Europe	Amlodipine vs Atenolol	9302	9228	40–78	40–78	≥2	279	350	3	3.8	High	Low
Dahlöf B et al 2005 [19]	Europe	Amlodipine vs Atenolol	9639(77)	9618(77)	63.0±8.5	63.0±8.5	≥5.5	327	422	3.4	4.4	High	Unclear
Turnbull F 2003 [23]	Europe	CCBs vs diuretic and β blocker	31031	37418	65	65	≥4	999	1358	3.2	3.6	High	Unclear
Black HR et al 2003 [28]	Europe	Verapamil vs Atenolol and	8179(43.8)	8297(44.2)	65.6±7.4	65.6±7.4	≥3	133	118	1.6	1.4	High	Unclear
		Hydrochlorothiazide											
Hansson L et al 1999 [29]	Europe	CCBs vs diuretic and β blocker	2196(34.0)	2213(32.0)	75.9	76	≥5	207	237	9.4	10.7	High	Low
Brown MJ et al 2000 [30]	Europe	Nifedipine vs Co-amilozide	3157(46.1)	3164(46.6)	55–80	55–80	≥3.5	67	74	2.1	2.3	High	Low
Pepine CJ et al 2003 [31]	Europe	Verapamil vs Atenolol	11267(48.1)	11309(47.7)	66.0±9.7	66.1±9.8	≥4	176	201	1.6	0.2	High	Low
Borhani NO et al 1996 [32]	Europe	Isradipine vs Hydrochlorothiazide	442(79.9)	441(75.7)	58.2±8.3	58.7±8.7	≥3	6	3	0.1	0.07	High	Low
Wang Y et al 1998 [24]	Asia	Nitrendipine vs Diuretics	141(62.4)	120(63.3)	56±11	54±13	≥5.1	0	3	0	2.5	High	Low
Hansson L et al 2000 [5]	Europe	Diltiazem vs Diuretic and β-blocker	5410(48.5)	5471(48.7)	60.5±6.5	60.3±6.5	≥4.5	159	196	2.9	3.6	High	Low
NICS-EH Study Group 1999 [3]	Asia	Nicardipine vs Trichlormethiazide	204(40.2)	210(26.2)	69.7±6.5	69.9±6.4	≥4.2	1	0	0.5	0	High	Low
Malacco E et al 2003 [33]	Europe	Lacidipine vs Chlorthalidone	942(39.6)	940(37.8)	72.3±7.5	72.4±7.6	≥5	37	38	4	4	Moderate	High
Ekbom T et al 2004 [18]	Europe	CCBs vs diuretic and β blocker	752(26.6)	756(21.8)	76.5	76.6	≥5	15	20	2	2.6	High	Low
Zanchetti A et al 2002 [22]	Europe	Lacidipine vs Atenolol	1177(54.2)	1157(55.4)	55.9±7.5	56.1±7.5	≥4	9	14	0.8	1.2	High	Low
Zanchetti A et al 1998 [34]	Europe	Verapamil vs Chlorthalidone	224(53.1)	232(51.3)	54.2±6.8	53.9±7.2	≥2	3	1	1.3	0.4	High	Unclear
Total			93111	105829				2795	3710	3	3.5		
													
**Overall**			**130466**	**143077**				**4145**	**5405**				

CCBs: Calcium Channel Blockers; ACEIs: Angiotensin-Converting Enzyme Inhibitors.

GRADE Working Group grades of evidence (see Supplementary Information online). High quality: Further research is very unlikely to change our confidence in the estimate of effect. Moderate quality: Further research is likely to have an important impact on our confidence in the estimate of effect and may change the estimate. Low quality: Further research is very likely to have an important impact on our confidence in the estimate of effect and is likely to change the estimate. Very low quality: We are very uncertain about the estimate.

The risk of bias assessment is done using RevMan (see Supplementary Information online). Low risk of bias: Plausible bias unlikely to seriously alter the results, low risk of bias for all key domains (within a study), and most information is from studies at low risk of bias (across studies). Unclear risk of bias: That raises some doubt about the results, unclear risk of bias for one or more key domains (within a study), and most information is from studies at low or unclear risk of bias (across studies). High risk of bias: Plausible bias that seriously weakens confidence in the results, high risk of bias for one or more key domains (within a study), the proportion of information from studies at high risk of bias is sufficient to affect the interpretation of results (across studies).

The issue of lost to follow-up or withdrew was identified as follows: 1) six studies [4], [24], [29], [32], [34], [37] reported that no patient was lost to follow-up or withdrew; 2) eight studies [14], [17]–[18], [23], [25], [35]–[36], [38] did not report the information of the patient’s follow-up or withdrew; 3) the remaining seventeen studies [3], [5], [12]–[13], [15]–[16], [19]–[22], [26]–[28], [30]–[31], [33], [39] reported that some patients were lost to follow-up or withdrew but not gave out the reasons, and the rate of lost to follow-up was not significantly difference between the experimental and control groups (see Supplementary Information online). Therefore, we did not perform the comparisons of incidence of withdrawals due to adverse effects for CCBs versus other drugs, because it was easy to result in a bias.

The results from the risk of bias assessment for each included study indicated that among the included thirty-one studies, fifteen reports [3], [5], [14], [17], [20]–[22], [27], [29]–[33], [37], [39] were low risk of bias, thirteen reports [4], [12], [13], [15], [18], [19], [23], [25], [28], [34]–[36], [38] were unclear risk of bias, and the remaining three studies [16], [26], [33] were high risk of bias (see Table 1 and Supplementary Information online).

There are two types of stroke, ischemic stroke (80%) and hemorrhagic stroke (20%). A total of 60–80% of hypertension patients (blood pressure >140/90 mmHg) face the risk of stroke. Hypertension is associated with ischemic- and hemorrhagic-stroke [40]. The detailed information of ischemic- or hemorrhagic-stroke was not presented in most original studies. The authors of included thirty-one studies have contact. Six reports authors could not contacted, nine reports authors did not response to us, five reports authors responded to us with the information we need, and eleven reports authors responded to us but did not give back the information we need. Therefore, we can not perform sub-groups analysis.

### Stroke Events of CCBs vs Placebo

**Figure 3:**
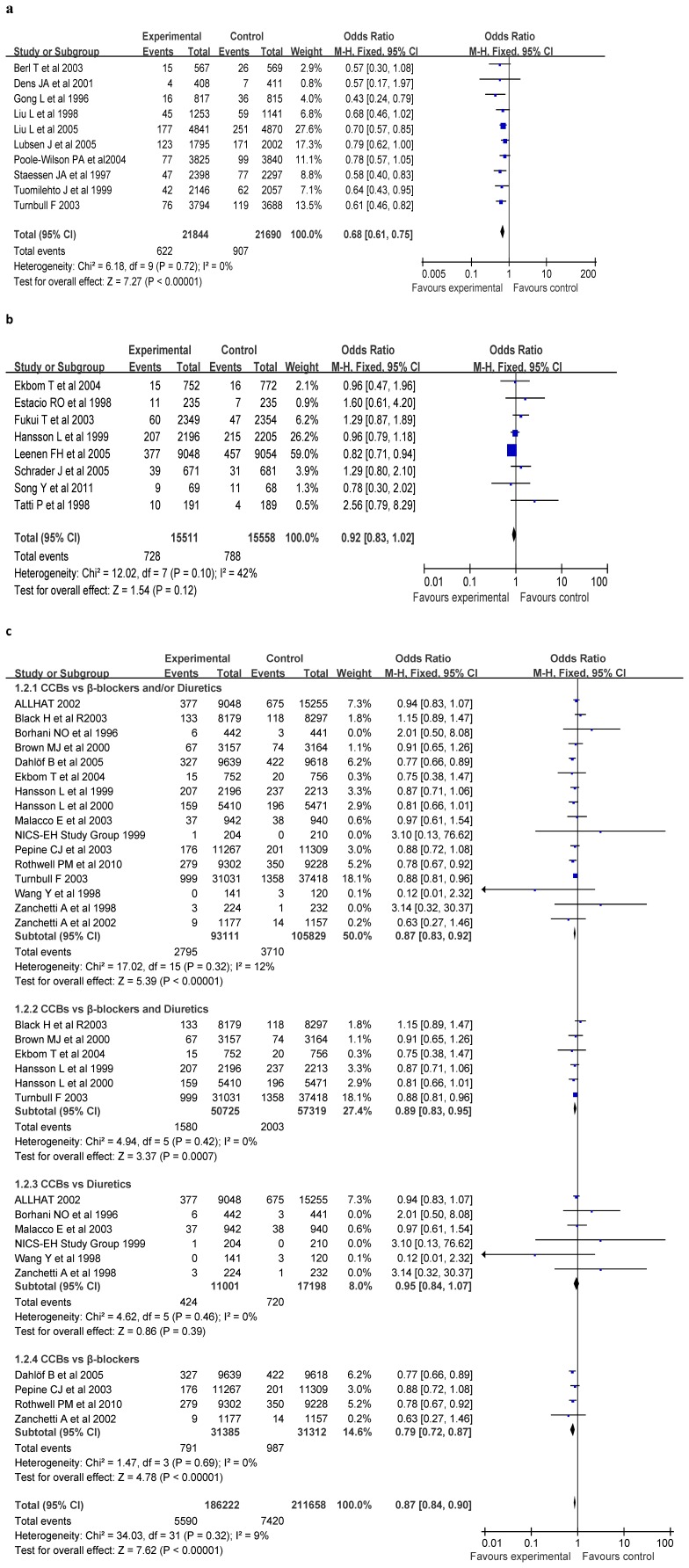
OR and 95% CI of individual studies and pooled data against stroke in the patients with hypertension. (**a**) Calcium channel blockers vs Placebo, (**b**) Calcium channel blockers vs ACEIs, and (**c**) Calcium channel blockers vs Diuretics or/and β Blockers.

Ten studies were included in this test [12], [14]–[16], [21], [23], [25]–[26], [37]–[38], which consisted of 21,844 experimental subjects and 21,690 controls, and 1,574 stroke events (622 in experimental group and 907 in control group). Statistic test revealed that the CCBs could significantly decline the stroke risk (OR = 0.68, 95% CI 0.61–0.75, p<1×10^−5^) compared with that of placebo (see Figure 3a). The incidence of stroke in CCBs group was decreased by 33.33% [(4.2%-2.8%) ÷ 4.2% × 100%] compared with that of placebo group (see Table 1).

### Stroke Events of CCBs vs ACEIs

Eight studies with a total of 15,511 experimental subjects and 15,558 controls were included in this analysis [4], [13], [18], [20], [29], [35]–[36], [39], and 1446 stroke events were reported (728 in experimental group and 788 in control group). No statistically significant difference was found (OR = 0.92, 95% CI 0.8–1.02, p = 0.12) between CCBs and ACEIs in their efficiency of against stroke (see Figure 3b). However, the incidence of stroke in CCBs group was decreased by 7.84% [(5.1%-4.7%) ÷ 5.1% × 100%] compared with that of ACEIs group (see Table 1).

### Stroke Events of CCBs vs Diuretics or/and β-adrenergic Blockers

Sixteen independent reports with 93,111 experimental subjects and 105,829 controls were included in this meta-analysis [3], [5], [17]–[19], [22]–[24], [27]–[34], which consisted of 6,505 stroke events (2795 in experimental group and 3710 in control group). The incidence of stroke in CCBs group was decreased by 14.28% [(3.5%-3%) ÷ 3.5% × 100%] compared with that of diuretics or/and β-adrenergic blockers group (see Table 1), and the CCBs were more effective (OR = 0.87, 95% CI 0.83–0.92, p<1×10^−5^) than diuretics or/and β blockers in the prevention of stroke (see Figure 3c). Results of subgroups analyses indicated that the CCBs were more effective than β-adrenergic blockers alone (OR = 0.79, 95% CI 0.72–0.87, p<1×10^−5^), β-adrenergic blockers combined with diuretics (OR = 0.89, 95% CI 0.83–0.95, p = 7×10^−5^), but not diuretics alone (OR = 0.95, 95% CI 0.84–1.07, p = 0.39) in the prevention of stroke (see Figure 3c).

## Discussion

This study demonstrates that the use of calcium channel blockers therapy, compared with placebo therapy (OR, 0.68), β-adrenergic blockers therapy (OR, 0.79), diuretics combined with β-adrenergic blockers therapy (OR, 0.89), angiotensin-converting enzyme inhibitors therapy (OR, 0.92), and diuretics therapy (OR, 0.95), was associated with a lower incidence of stroke events in the patients with hypertension. In this combined study of different hypertension populations, the risk of stroke events reduction for patients receiving calcium channel blockers therapy was similar. Due to different sample size in different treatment groups, it is essential to use and interpret the above results with cautions. These findings present new evidence to support the idea that the CCBs reduced stroke more than placebo and β-adrenergic blockers but were not different than ACEIs and diuretics. Hypertension is only one of the major risk factors in the development of stroke, the number of stroke events remains high even though the antihypertensive agents are used extensively [39]. Therefore, other risk factors or/and the biological processes underlying the pathophysiology of stroke warrant further studies in the near future.

The findings of our work indicated that CCBs reduced stroke more than placebo and β-adrenergic blockers, but the detail molecular mechanisms are not well known and remain to be excavated in the future. By now, it can be explained in part by that CCBs can generate stronger antihypertensive effect (by dilating the blood vessels) than that of beta-blockers (by reducing the blood flow of cardiac output) or that of placebo (by confounders). These results reported here provide strong evidence linking controlling hypertension to a reduced risk of stroke. Meanwhile, this study has some limitations and caveats. First, as not all clinical data were available from each original report, other subclasses-stratified analyses could not be performed; the risk of bias assessment in this work could rob the credibility of results. Second, only studies reported in English or Chinese were included, which might be vulnerable to the bias of language and ethnicity. Third, the whole sample size in this study is sufficient for statistic purposes, but the sample size of each subgroup is relatively small and susceptible to false positive or negative results. Fourth, after the treatment of antihypertensive agents, the years of followed-up between studies varied greatly (from 1 to 5.5 years), which also could result in a bias. Finally, only four kinds of antihypertensive agents were tested in this report; addition of other drugs and withdrawals of treatment may also lead to an underestimation of the real differences in stroke risk between the previous reports. Further studies are required to investigate the association between other antihypertensive agents and stroke-risk, and to provide a better estimate the benefits of antihypertensive agents against stroke in the hypertension populations.

In conclusions, the present analysis shows that CCBs, ACEIs, diuretics, and β-adrenergic blockers can decline the incidence of stroke in the hypertension populations. Among them, CCBs reduced stroke more than placebo and β-adrenergic blockers, but were not different than ACEIs and diuretics. More head to head RCTs are warranted. This systematic review and meta-analysis provides a thorough examination of the literature on the effect of first-line antihypertensive agents in the prevention of stroke, and provide a foundation of knowledge on which clinical and public health messaging deserves to be further discussed.

## Supporting Information

Supplementary Information S1 The quality assessment of evidence by GRADEprofiler.
(DOC)

Supplementary Information S2 The risk of bias assessment by RevMan.
(DOC)
