# Supplementary material for: The Effects of Calcium Channel Blockers in the Prevention of Stroke in Adults with Hypertension: A Meta-Analysis of Data from 273,543 Participants in 31 Randomized Controlled Trials
Source: PLoS One. 2013 Mar 6;8(3):e57854. doi: 10.1371/journal.pone.0057854 (PMC3590278; doi:10.1371/journal.pone.0057854)
Supplement: Supplementary Information S1 — The quality assessment of evidence by GRADEprofiler. (DOC) [file pone.0057854.s001.doc]

**Supplementary Information 1**

**The quality assessment of evidence for each included study by GRADEprofiler software version 3.2.2.**

| **CCBs vs Placebo for reducing the incidence of stroke** | | | | | | |
| --- | --- | --- | --- | --- | --- | --- |
| **Patient or population:** patients with hypertension  **Settings:** patients  **Intervention(experimental group):** CCBs  **Comparison(control group):** Placebo | | | | | | |
| **Outcomes** | **Illustrative comparative risks* (95% CI)** | | **Relative effect (95% CI)** | **No of Participants (studies)** | **Quality of the evidence (GRADE)** | **Comments** |
|  | Assumed risk | Corresponding risk |  |  |  |  |
|  | **Placebo** | **CCBs** |  |  |  | **(patients of lost to follow-up)** |
| **Poole-Wilson PA et al 2004 [16]** Follow-up: 4.9 years | **26 per 1000** | **20 per 1000** (15 to 27) | **OR 0.78**  (0.57 to 1.05) | 7665 (1 study) | ⊕⊕⊕⊝ **moderate**^2^ | 491 lost in experimental group  470 lost in control group |
| **Lubsen J et al 2005 [14]** Follow-up: 5.5 years | **85 per 1000** | **68 per 1000** (54 to 85) | **OR 0.79**  (0.62 to 1.00) | 3797 (1 study) | ⊕⊕⊕⊕ **high**^1^ | No report |
| **Turnbull F 2003 [23]** Follow-up: 4 years | **32 per 1000** | **20 per 1000** (15 to 26) | **OR 0.61**  (0.46 to 0.82) | 7482 (1 study) | ⊕⊕⊕⊕ **high**^1^ | No report |
| **Liu L et al 2005 [21]** Follow-up: 3.5 years | **52 per 1000** | **37 per 1000** (30 to 45) | **OR 0.70**  (0.57 to 0.85) | 9711 (1 study) | ⊕⊕⊕⊕ **high**^1^ | 14 lost in experimental group  16 lost in control group |
| **Berl T et al 2003 [37]** Follow-up: 2.6 years | **46 per 1000** | **27 per 1000** (14 to 49) | **OR 0.57**  (0.3 to 1.08) | 1136 (1 study) | ⊕⊕⊕⊕ **high**^1^ | No patient was lost to follow-up and withdrew |
| **Tuomilehto J et al 1999 [38]** Follow-up: 2 years | **30 per 1000** | **19 per 1000** (13 to 29) | **OR 0.64**  (0.43 to 0.95) | 4203 (1 study) | ⊕⊕⊕⊕ **high**^1^ | No report. |
| **Dens JA et al 2001 [25]** Follow-up: 3 years | **17 per 1000** | **10 per 1000** (3 to 33) | **OR 0.57**  (0.17 to 1.97) | 819 (1 study) | ⊕⊕⊕⊕ **high**^1^ | No report. |
| **Gong L et al 1996 [26]** Follow-up: 2.5 years | **44 per 1000** | **19 per 1000** (11 to 35) | **OR 0.43**  (0.24 to 0.79) | 1632 (1 study) | ⊕⊕⊕⊝ **moderate**^2^ | 134 lost in experimental group  174 lost in control group |
| **Liu L et al 1998 [15]** Follow-up: 4 years | **52 per 1000** | **36 per 1000** (25 to 53) | **OR 0.68**  (0.46 to 1.02) | 2394 (1 study) | ⊕⊕⊕⊕ **high**^1^ | 115 lost in experimental group  122 lost in control group |
| **Staessen JA et al 1997 [12]** Follow-up: 2 years | **34 per 1000** | **20 per 1000** (14 to 28) | **OR 0.58**  (0.4 to 0.83) | 4695 (1 study) | ⊕⊕⊕⊕ **high**^1^ | 121 lost in experimental group  116 lost in control group |
| *The basis for the **assumed risk** (e.g. the median control group risk across studies) is provided in footnotes. The **corresponding risk** (and its 95% confidence interval) is based on the assumed risk in the comparison group and the **relative effect** of the intervention (and its 95% CI).  **CI:** Confidence interval; **OR:** Odds ratio; | | | | | | |
| GRADE Working Group grades of evidence  **High quality:** Further research is very unlikely to change our confidence in the estimate of effect.  **Moderate quality:** Further research is likely to have an important impact on our confidence in the estimate of effect and may change the estimate.  **Low quality:** Further research is very likely to have an important impact on our confidence in the estimate of effect and is likely to change the estimate.  **Very low quality:** We are very uncertain about the estimate. | | | | | | |

The trial conducted a sensitivity analysis and it was found that a small change of incidence rates and loss-to-follow-up led to only a small reduction of statistical power (70 –80%):

^1^ The number of lost to follow-up and withdrawals was less than 10% defined as high quality; and the rate of lost to follow-up was not significantly difference between the experimental and control groups.

^2^ The number of lost to follow-up and withdrawals was more than 10% defined as moderate quality; and the rate of lost to follow-up was not significantly difference between the experimental and control groups.

**CCBs vs Placebo**

| **Quality assessment** | | | | | | | **Summary of findings** | | | | | **Importance** |
| --- | --- | --- | --- | --- | --- | --- | --- | --- | --- | --- | --- | --- |
|  |  |  |  |  |  |  | **No of patients** | | **Effect** | | **Quality** |  |
| **No of studies** | **Design** | **Limitations** | **Inconsistency** | **Indirectness** | **Imprecision** | **Other considerations** | **CCBs** | **Placebo** | **Relative (95% CI)** | **Absolute** |  |  |
| **Poole-Wilson PA et al 2004 [16] (follow-up 4.9 years)** | | | | | | | | | | | | |
| 1 | randomised trials | no serious limitations | no serious inconsistency | no serious indirectness | no serious imprecision | none | 77/3825 (2%) | 99/3840 (2.6%) | OR 0.78 (0.57 to 1.05) | 6 fewer per 1000 (from 11 fewer to 1 more) | ⊕⊕⊕O MODERATE | IMPORTANT |
| **Lubsen J et al 2005 [14] (follow-up 5.5 years)** | | | | | | | | | | | | |
| 1 | randomised trials | no serious limitations | no serious inconsistency | no serious indirectness | no serious imprecision | none | 123/1795 (6.9%) | 171/2002 (8.5%) | OR 0.79 (0.62 to 1.00) | 17 fewer per 1000 (from 31 fewer to 0 more) | ⊕⊕⊕⊕ HIGH | IMPORTANT |
| **Turnbull F 2003 [23] (follow-up 4 years)** | | | | | | | | | | | | |
| 1 | randomised trials | no serious limitations | no serious inconsistency | no serious indirectness | no serious imprecision | none | 76/3794 (2%) | 119/3688 (3.2%) | OR 0.61 (0.46 to 0.82) | 12 fewer per 1000 (from 6 fewer to 17 fewer) | ⊕⊕⊕⊕ HIGH | IMPORTANT |
| **Liu L et al 2005 [21] (follow-up 3.5 years)** | | | | | | | | | | | | |
| 1 | randomised trials | no serious limitations | no serious inconsistency | no serious indirectness | no serious imprecision | none | 177/4841 (3.7%) | 251/4870 (5.2%) | OR 0.70 (0.57 to 0.85) | 15 fewer per 1000 (from 7 fewer to 21 fewer) | ⊕⊕⊕⊕ HIGH | IMPORTANT |
| **Berl T et al 2003 [37] (follow-up 2.6 years)** | | | | | | | | | | | | |
| 1 | randomised trials | no serious limitations | no serious inconsistency | no serious indirectness | no serious imprecision | none | 15/567 (2.6%) | 26/569 (4.6%) | OR 0.57 (0.3 to 1.08) | 19 fewer per 1000 (from 32 fewer to 3 more) | ⊕⊕⊕⊕ HIGH | IMPORTANT |
| **Tuomilehto J et al 1999 [38] (follow-up 2 years)** | | | | | | | | | | | | |
| 1 | randomised trials | no serious limitations | no serious inconsistency | no serious indirectness | no serious imprecision | none | 42/2146 (2%) | 62/2057 (3%) | OR 0.64 (0.43 to 0.95) | 11 fewer per 1000 (from 1 fewer to 17 fewer) | ⊕⊕⊕⊕ HIGH | IMPORTANT |
| **Dens JA et al 2001 [25] (follow-up 3 years)** | | | | | | | | | | | | |
| 1 | randomised trials | no serious limitations | no serious inconsistency | no serious indirectness | no serious imprecision | none | 4/408 (1%) | 7/411 (1.7%) | OR 0.57 (0.17 to 1.97) | 7 fewer per 1000 (from 14 fewer to 16 more) | ⊕⊕⊕⊕ HIGH | IMPORTANT |
| **Gong L et al 1996 [26] (follow-up 2.5 years)** | | | | | | | | | | | | |
| 1 | randomised trials | no serious limitations | no serious inconsistency | no serious indirectness | no serious imprecision | none | 16/817 (2%) | 36/815 (4.4%) | OR 0.43 (0.24 to 0.79) | 25 fewer per 1000 (from 9 fewer to 33 fewer) | ⊕⊕⊕O MODERATE | IMPORTANT |
| **Liu L et al 1998 [15] (follow-up 4 years)** | | | | | | | | | | | | |
| 1 | randomised trials | no serious limitations | no serious inconsistency | no serious indirectness | no serious imprecision | none | 45/1253 (3.6%) | 59/1141 (5.2%) | OR 0.68 (0.46 to 1.02) | 16 fewer per 1000 (from 27 fewer to 1 more) | ⊕⊕⊕⊕ HIGH | IMPORTANT |
| **Staessen JA et al 1997 [12] (follow-up 2 years)** | | | | | | | | | | | | |
| 1 | randomised trials | no serious limitations | no serious inconsistency | no serious indirectness | no serious imprecision | none | 47/2398 (2%) | 77/2297 (3.4%) | OR 0.58 (0.4 to 0.83) | 14 fewer per 1000 (from 6 fewer to 20 fewer) | ⊕⊕⊕⊕ HIGH | IMPORTANT |

| **CCBs vs ACEIs for reduce the incidence of stroke** | | | | | | |
| --- | --- | --- | --- | --- | --- | --- |
| **Patient or population:** patients with hypertension  **Settings:** patients  **Intervention(experimental group):** CCBs  **Comparison(control group):** ACEIs | | | | | | |
| **Outcomes** | **Illustrative comparative risks* (95% CI)** | | **Relative effect (95% CI)** | **No of Participants (studies)** | **Quality of the evidence (GRADE)** | **Comments** |
|  | Assumed risk | Corresponding risk |  |  |  |  |
|  | **ACEIs** | **CCBs** |  |  |  | **(patients of lost to follow-up)** |
| **Estacio RO et al 1998 [35]** Follow-up: 5 years | **30 per 1000** | **47 per 1000** (19 to 115) | **OR 1.60**  (0.61 to 4.20) | 470 (1 study) | ⊕⊕⊕⊕ **high^1^** | No report |
| **Leenen FH et al 2005 [20]** Follow-up: 4 years | **50 per 1000** | **41 per 1000** (36 to 47) | **OR 0.82**  (0.71 to 0.94) | 18102 (1 study) | ⊕⊕⊕⊕ **high^1^** | 258 lost in experimental group  276 lost in control group |
| **Fukui T et al 2003 [36]** Follow-up: 3.2 years | **20 per 1000** | **26 per 1000** (17 to 37) | **OR 1.29**  (0.87 to 1.89) | 4703 (1 study) | ⊕⊕⊕⊕ **high^1^** | No report |
| **Song Y et al 2011 [4]** Follow-up: 1 year | **162 per 1000** | **131 per 1000** (55 to 281) | **OR 0.78**  (0.30 to 2.02) | 137 (1 study) | ⊕⊕⊕⊕ **high^1^** | No patient was lost to follow-up and withdrew |
| **Tatti P et al 1998 [13]** Follow-up: 3.5 years | **21 per 1000** | **52 per 1000** (17 to 151) | **OR 2.56**  (0.79 to 8.29) | 380 (1 study) | ⊕⊕⊕⊕ **high^1^** | 3 lost in experimental group  1 lost in control group |
| **Hansson L et al 1999 [29]** Follow-up: 5 years | **98 per 1000** | **94 per 1000** (79 to 114) | **OR 0.96**  (0.79 to 1.18) | 4401 (1 study) | ⊕⊕⊕⊕ **high^1^** | No patient was lost to follow-up and withdrew |
| **Schrader J et al 2005 [39]** Follow-up: 2.5 years | **46 per 1000** | **59 per 1000** (37 to 92) | **OR 1.29**  (0.80 to 2.10) | 1352 (1 study) | ⊕⊕⊕⊕ **high^1^** | 12 lost in experimental group  14 lost in control group |
| **Ekbom T et al 2004 [18]** Follow-up: 5 years | **21 per 1000** | **20 per 1000** (10 to 40) | **OR 0.96**  (0.47 to 1.96) | 1524 (1 study) | ⊕⊕⊕⊕ **high^1^** | No report |
| *The basis for the **assumed risk** (e.g. the median control group risk across studies) is provided in footnotes. The **corresponding risk** (and its 95% confidence interval) is based on the assumed risk in the comparison group and the **relative effect** of the intervention (and its 95% CI).  **CI:** Confidence interval; **OR:** Odds ratio; | | | | | | |
| GRADE Working Group grades of evidence  **High quality:** Further research is very unlikely to change our confidence in the estimate of effect.  **Moderate quality:** Further research is likely to have an important impact on our confidence in the estimate of effect and may change the estimate.  **Low quality:** Further research is very likely to have an important impact on our confidence in the estimate of effect and is likely to change the estimate.  **Very low quality:** We are very uncertain about the estimate. | | | | | | |

The trial conducted a sensitivity analysis and it was found that a small change of incidence rates and loss-to-follow-up led to only a small reduction of statistical power (70 –80%):

^1^ The number of lost to follow-up and withdrawals was less than 10% defined as high quality; and the rate of lost to follow-up was not significantly difference between the experimental and control groups.

^2^ The number of lost to follow-up and withdrawals was more than 10% defined as moderate quality; and the rate of lost to follow-up was not significantly difference between the experimental and control groups.

**CCBs vs ACEIs**

| **Quality assessment** | | | | | | | **Summary of findings** | | | | | **Importance** |
| --- | --- | --- | --- | --- | --- | --- | --- | --- | --- | --- | --- | --- |
|  |  |  |  |  |  |  | **No of patients** | | **Effect** | | **Quality** |  |
| **No of studies** | **Design** | **Limitations** | **Inconsistency** | **Indirectness** | **Imprecision** | **Other considerations** | **CCBs** | **ACEIs** | **Relative (95% CI)** | **Absolute** |  |  |
| **Estacio RO et al 1998 [35] (follow-up 5 years)** | | | | | | | | | | | | |
| 1 | randomised trials | no serious limitations | no serious inconsistency | no serious indirectness | no serious imprecision | none | 11/235 (4.7%) | 7/235 (3%) | OR 1.60 (0.61 to 4.20) | 17 more per 1000 (from 11 fewer to 84 more) | ⊕⊕⊕⊕ HIGH | IMPORTANT |
| **Leenen FH et al 2005 [20] (follow-up 4 years)** | | | | | | | | | | | | |
| 1 | randomised trials | no serious limitations | no serious inconsistency | no serious indirectness | no serious imprecision | none | 377/9048 (4.2%) | 457/9054 (5%) | OR 0.82 (0.71 to 0.94) | 9 fewer per 1000 (from 3 fewer to 14 fewer) | ⊕⊕⊕⊕ HIGH | IMPORTANT |
| **Fukui T et al 2003 [36] (follow-up 3.2 years)** | | | | | | | | | | | | |
| 1 | randomised trials | no serious limitations | no serious inconsistency | no serious indirectness | no serious imprecision | none | 60/2349 (2.6%) | 47/2354 (2%) | OR 1.29 (0.87 to 1.89) | 6 more per 1000 (from 3 fewer to 17 more) | ⊕⊕⊕⊕ HIGH | IMPORTANT |
| **Song Y et al 2011 [4] (follow-up 1)** | | | | | | | | | | | | |
| 1 | randomised trials | no serious limitations | no serious inconsistency | no serious indirectness | no serious imprecision | reporting bias^1^ | 9/69 (13%) | 11/68 (16.2%) | OR 0.78 (0.30 to 2.02) | 31 fewer per 1000 (from 107 fewer to 119 more) | ⊕⊕⊕⊕ HIGH | NOT IMPORTANT |
| **Tatti P et al 1998 [13] (follow-up 3.5 years)** | | | | | | | | | | | | |
| 1 | randomised trials | no serious limitations | no serious inconsistency | no serious indirectness | no serious imprecision | none | 10/191 (5.2%) | 4/189 (2.1%) | OR 2.56 (0.79 to 8.29) | 31 more per 1000 (from 4 fewer to 131 more) | ⊕⊕⊕⊕ HIGH | IMPORTANT |
| **Hansson L et al 1999 [29] (follow-up 5 years)** | | | | | | | | | | | | |
| 1 | randomised trials | no serious limitations | no serious inconsistency | no serious indirectness | no serious imprecision | none | 207/2196 (9.4%) | 215/2205 (9.8%) | OR 0.96 (0.79 to 1.18) | 4 fewer per 1000 (from 19 fewer to 16 more) | ⊕⊕⊕⊕ HIGH | IMPORTANT |
| **Schrader J et al 2005 [39] (follow-up 2.5 years)** | | | | | | | | | | | | |
| 1 | randomised trials | no serious limitations | no serious inconsistency | no serious indirectness | no serious imprecision | none | 39/671 (5.8%) | 31/681 (4.6%) | OR 1.29 (0.80 to 2.10) | 12 more per 1000 (from 9 fewer to 46 more) | ⊕⊕⊕⊕ HIGH | IMPORTANT |
| **Ekbom T et al 2004 [18] (follow-up 5 years)** | | | | | | | | | | | | |
| 1 | randomised trials | no serious limitations | no serious inconsistency | no serious indirectness | no serious imprecision | none | 15/752 (2%) | 16/772 (2.1%) | OR 0.96 (0.47 to 1.96) | 1 fewer per 1000 (from 11 fewer to 19 more) | ⊕⊕⊕⊕ HIGH | IMPORTANT |

| **CCBs compared to β blockers or/and Diuretics for hypertension** | | | | | | |
| --- | --- | --- | --- | --- | --- | --- |
| **Patient or population:** patients with hypertension  **Settings:** patients.  **Intervention(experimental group):** CCBs  **Comparison(control group):** β blockers or/and Diuretics | | | | | | |
| **Outcomes** | **Illustrative comparative risks* (95% CI)** | | **Relative effect (95% CI)** | **No of Participants (studies)** | **Quality of the evidence (GRADE)** | **Comments** |
|  | Assumed risk | Corresponding risk |  |  |  |  |
|  | **β blockers or/and Diuretics** | **CCBs** |  |  |  | **(patients of lost to follow-up)p65filere, version 6.1083.5%for experimental group and 78.6%for controlled group at tow year000000000000000000000000000000000000** |
| **ALLHAT 2002 [27]** Follow-up: 4.9 years | **44 per 1000** | **41 per 1000** (37 to 47) | **OR 0.94**  (0.83 to 1.07) | 24303 (1 study) | ⊕⊕⊕⊕ **high**^1^ | 258 lost in experimental group  419 lost in control group |
| **Rothwell PM et al 2010 [17]** Follow-up: 2 years | **38 per 1000** | **30 per 1000** (26 to 35) | **OR 0.78**  (0.67 to 0.92) | 18530 (1 study) | ⊕⊕⊕⊕ **high**^1^ | No report |
| **Dahlöf B et al 2005 [19]** Follow-up: 5.5 years | **44 per 1000** | **34 per 1000** (29 to 39) | **OR 0.77**  (0.66 to 0.89) | 19257 (1 study) | ⊕⊕⊕⊕ **high**^1^ | 121 lost in experimental group  171 lost in control group |
| **Turnbull F 2003 [23]** Follow-up: 4 years | **36 per 1000** | **32 per 1000** (29 to 35) | **OR 0.88**  (0.81 to 0.96) | 68449 (1 study) | ⊕⊕⊕⊕ **high**^1^ | No report |
| **Black HR et al 2003 [28]** Follow-up: 3 years | **14 per 1000** | **16 per 1000** (12 to 20) | **OR 1.15**  (0.89 to 1.47) | 16476 (1 study) | ⊕⊕⊕⊕ **high**^1^ | 570 lost in experimental group  563 lost in control group |
| **Hansson L et al 1999 [29]** Follow-up: 5 years | **107 per 1000** | **94 per 1000** (78 to 113) | **OR 0.87**  (0.71 to 1.06) | 4409 (1 study) | ⊕⊕⊕⊕ **high**^1^ | No patient was lost to follow-up and withdrew |
| **Brown MJ et al 2000 [30]** Follow-up: 3.5 years | **23 per 1000** | **21 per 1000** (15 to 29) | **OR 0.91**  (0.65 to 1.26) | 6321 (1 study) | ⊕⊕⊕⊕ **high**^1^ | 66 lost in experimental group  83 lost in control group |
| **Pepine CJ et al 2003 [31]** Follow-up: 4 years | **18 per 1000** | **16 per 1000** (13 to 19) | **OR 0.88**  (0.72 to 1.08) | 22576 (1 study) | ⊕⊕⊕⊕ **high**^1^ | 300 lost in experimental group  268 lost in control group |
| **Borhani NO et al 1996 [32]** Follow-up: 3 years | **7 per 1000** | **14 per 1000** (4 to 54) | **OR 2.01**  (0.50 to 8.08) | 883 (1 study) | ⊕⊕⊕⊕ **high**^1^ | No patient was lost to follow-up and withdrew |
| **Wang Y et al 1998 [24]** Follow-up: 5.1 years | **25 per 1000** | **3 per 1000** (0 to 56) | **OR 0.12**  (0.01 to 2.32) | 261 (1 study) | ⊕⊕⊕⊕ **high**^1^ | No patient was lost to follow-up and withdrew |
| **Hansson L et al 2000 [5]** Follow-up: 4.5 years | **36 per 1000** | **29 per 1000** (24 to 36) | **OR 0.81**  (0.66 to 1.01) | 10881 (1 study) | ⊕⊕⊕⊕ **high**^1^ | 24 lost in experimental group  28 lost in control group |
| **NICS-EH Study Group 1999 [3]** Follow-up: 4.2 years |  |  | **OR 3.10**  (0.13 to 76.62) | 414 (1 study) | ⊕⊕⊕⊕ **high**^1^ | 6 lost in experimental group  9 lost in control group |
| **Malacco E et al 2003 [33]** Follow-up: 5 years | **40 per 1000** | **39 per 1000** (25 to 60) | **OR 0.97**  (0.61 to 1.54) | 1882 (1 study) | ⊕⊕⊕⊝ **moderate**^2^ | 116 lost in experimental group  104 lost in control group |
| **Ekbom T et al 2004 [18]** Follow-up: 5 years | **26 per 1000** | **20 per 1000** (10 to 38) | **OR 0.75**  (0.38 to 1.47) | 1508 (1 study) | ⊕⊕⊕⊕ **high**^1^ | No report |
| **Zanchetti A et al 2002 [22]** Follow-up: 4 years | **12 per 1000** | **8 per 1000** (3 to 17) | **OR 0.63**  (0.27 to 1.46) | 2334 (1 study) | ⊕⊕⊕⊕ **high**^1^ | 49 lost in experimental group  43 lost in control group |
| **Zanchetti A et al 1998 [34]** Follow-up: 2 years | **4 per 1000** | **12 per 1000** (1 to 109) | **OR 3.14**  (0.32 to 30.37) | 456 (1 study) | ⊕⊕⊕⊕ **high**^1^ | No patient was lost to follow-up and withdrew |
| *The basis for the **assumed risk** (e.g. the median control group risk across studies) is provided in footnotes. The **corresponding risk** (and its 95% confidence interval) is based on the assumed risk in the comparison group and the **relative effect** of the intervention (and its 95% CI).  **CI:** Confidence interval; **OR:** Odds ratio; | | | | | | |
| GRADE Working Group grades of evidence  **High quality:** Further research is very unlikely to change our confidence in the estimate of effect.  **Moderate quality:** Further research is likely to have an important impact on our confidence in the estimate of effect and may change the estimate.  **Low quality:** Further research is very likely to have an important impact on our confidence in the estimate of effect and is likely to change the estimate.  **Very low quality:** We are very uncertain about the estimate. | | | | | | |

The trial conducted a sensitivity analysis and it was found that a small change of incidence rates and loss-to-follow-up led to only a small reduction of statistical power (70 –80%):

^1^ The number of lost to follow-up and withdrawals was less than 10% defined as high quality; and the rate of lost to follow-up was not significantly difference between the experimental and control groups.

^2^ The number of lost to follow-up and withdrawals was more than 10% defined as moderate quality; and the rate of lost to follow-up was not significantly difference between the experimental and control groups.

**CCBs vsβ blockers or/and Diuretics**

| **Quality assessment** | | | | | | | **Summary of findings** | | | | | **Importance** |
| --- | --- | --- | --- | --- | --- | --- | --- | --- | --- | --- | --- | --- |
|  |  |  |  |  |  |  | **No of patients** | | **Effect** | | **Quality** |  |
| **No of studies** | **Design** | **Limitations** | **Inconsistency** | **Indirectness** | **Imprecision** | **Other considerations** | **CCBs** | **β blockers or/and Diuretics** | **Relative (95% CI)** | **Absolute** |  |  |
| **ALLHAT 2002 [27] (follow-up 4.9 years)** | | | | | | | | | | | | |
| 1 | randomised trials | no serious limitations | no serious inconsistency | no serious indirectness | no serious imprecision | none | 377/9048 (4.2%) | 675/15255 (4.4%) | OR 0.94 (0.83 to 1.07) | 3 fewer per 1000 (from 7 fewer to 3 more) | ⊕⊕⊕⊕ HIGH | IMPORTANT |
| **Rothwell PM et al 2010 [17] (follow-up 2 years)** | | | | | | | | | | | | |
| 1 | randomised trials | no serious limitations | no serious inconsistency | no serious indirectness | no serious imprecision | none | 279/9302 (3%) | 350/9228 (3.8%) | OR 0.78 (0.67 to 0.92) | 8 fewer per 1000 (from 3 fewer to 12 fewer) | ⊕⊕⊕⊕ HIGH | IMPORTANT |
| **Dahlöf B et al 2005 [19] (follow-up 5.5 years)** | | | | | | | | | | | | |
| 1 | randomised trials | no serious limitations | no serious inconsistency | no serious indirectness | no serious imprecision | none | 327/9639 (3.4%) | 422/9618 (4.4%) | OR 0.77 (0.66 to 0.89) | 10 fewer per 1000 (from 5 fewer to 14 fewer) | ⊕⊕⊕⊕ HIGH | IMPORTANT |
| **Turnbull F 2003 [23] (follow-up 4 years)** | | | | | | | | | | | | |
| 1 | randomised trials | no serious limitations | no serious inconsistency | no serious indirectness | no serious imprecision | none | 999/31031 (3.2%) | 1358/37418 (3.6%) | OR 0.88 (0.81 to 0.96) | 4 fewer per 1000 (from 1 fewer to 7 fewer) | ⊕⊕⊕⊕ HIGH | IMPORTANT |
| **Black HR et al 2003 [28] (follow-up 3 years)** | | | | | | | | | | | | |
| 1 | randomised trials | no serious limitations | no serious inconsistency | no serious indirectness | no serious imprecision | none | 133/8179 (1.6%) | 118/8297 (1.4%) | OR 1.15 (0.89 to 1.47) | 2 more per 1000 (from 2 fewer to 7 more) | ⊕⊕⊕⊕ HIGH | IMPORTANT |
| **Hansson L et al 1999 [29] (follow-up 5 years)** | | | | | | | | | | | | |
| 1 | randomised trials | no serious limitations | no serious inconsistency | no serious indirectness | no serious imprecision | none | 207/2196 (9.4%) | 237/2213 (10.7%) | OR 0.87 (0.71 to 1.06) | 13 fewer per 1000 (from 29 fewer to 6 more) | ⊕⊕⊕⊕ HIGH | IMPORTANT |
| **Brown MJ et al 2000 [30] (follow-up 3.5 years)** | | | | | | | | | | | | |
| 1 | randomised trials | no serious limitations | no serious inconsistency | no serious indirectness | no serious imprecision | none | 67/3157 (2.1%) | 74/3164 (2.3%) | OR 0.91 (0.65 to 1.26) | 2 fewer per 1000 (from 8 fewer to 6 more) | ⊕⊕⊕⊕ HIGH | IMPORTANT |
| **Pepine CJ et al 2003 [31] (follow-up 4 years)** | | | | | | | | | | | | |
| 1 | randomised trials | no serious limitations | no serious inconsistency | no serious indirectness | no serious imprecision | none | 176/11267 (1.6%) | 201/11309 (1.8%) | OR 0.88 (0.72 to 1.08) | 2 fewer per 1000 (from 5 fewer to 1 more) | ⊕⊕⊕⊕ HIGH | IMPORTANT |
| **Borhani NO et al 1996 [32] (follow-up 3 years)** | | | | | | | | | | | | |
| 1 | randomised trials | no serious limitations | no serious inconsistency | no serious indirectness | no serious imprecision | none | 6/442 (1.4%) | 3/441 (0.7%) | OR 2.01 (0.50 to 8.08) | 7 more per 1000 (from 3 fewer to 46 more) | ⊕⊕⊕⊕ HIGH | IMPORTANT |
| **Wang Y et al 1998 [24] (follow-up 5.1 years)** | | | | | | | | | | | | |
| 1 | randomised trials | no serious limitations | no serious inconsistency | no serious indirectness | no serious imprecision | none | 0/141 (0%) | 3/120 (2.5%) | OR 0.12 (0.01 to 2.32) | 22 fewer per 1000 (from 25 fewer to 31 more) | ⊕⊕⊕⊕ HIGH | IMPORTANT |
| **Hansson L et al 2000 [5] (follow-up 4.5 years)** | | | | | | | | | | | | |
| 1 | randomised trials | no serious limitations | no serious inconsistency | no serious indirectness | no serious imprecision | none | 159/5410 (2.9%) | 196/5471 (3.6%) | OR 0.81 (0.66 to 1.01) | 7 fewer per 1000 (from 12 fewer to 0 more) | ⊕⊕⊕⊕ HIGH | IMPORTANT |
| **NICS-EH Study Group 1999 [3] (follow-up 4.2 years)** | | | | | | | | | | | | |
| 1 | randomised trials | no serious limitations | no serious inconsistency | no serious indirectness | no serious imprecision | none | 1/204 (0.5%) | 0/210 (0%) | OR 3.10 (0.13 to 76.62) | 0 more per 1000 (from 0 fewer to 0 more) | ⊕⊕⊕⊕ HIGH | IMPORTANT |
| **Malacco E et al 2003 [33] (follow-up 5 years)** | | | | | | | | | | | | |
| 1 | randomised trials | no serious limitations | no serious inconsistency | no serious indirectness | no serious imprecision | none | 37/942 (3.9%) | 38/940 (4%) | OR 0.97 (0.61 to 1.54) | 1 fewer per 1000 (from 15 fewer to 20 more) | ⊕⊕⊕O MODERATE | IMPORTANT |
| **Ekbom T et al 2004 [18] (follow-up 5 years)** | | | | | | | | | | | | |
| 1 | randomised trials | no serious limitations | no serious inconsistency | no serious indirectness | no serious imprecision | none | 15/752 (2%) | 20/756 (2.6%) | OR 0.75 (0.38 to 1.47) | 6 fewer per 1000 (from 16 fewer to 12 more) | ⊕⊕⊕⊕ HIGH | IMPORTANT |
| **Zanchetti A et al 2002 [22] (follow-up 4 years)** | | | | | | | | | | | | |
| 1 | randomised trials | no serious limitations | no serious inconsistency | no serious indirectness | no serious imprecision | none | 9/1177 (0.8%) | 14/1157 (1.2%) | OR 0.63 (0.27 to 1.46) | 4 fewer per 1000 (from 9 fewer to 5 more) | ⊕⊕⊕⊕ HIGH | IMPORTANT |
| **Zanchetti A et al 1998 [34] (follow-up 2 years)** | | | | | | | | | | | | |
| 1 | randomised trials | no serious limitations | no serious inconsistency | no serious indirectness | no serious imprecision | none | 3/224 (1.3%) | 1/232 (0.4%) | OR 3.14 (0.32 to 30.37) | 9 more per 1000 (from 3 fewer to 112 more) | ⊕⊕⊕⊕ HIGH | IMPORTANT |

**The quality assessment of evidence for each included subgroup by GRADEprofiler software version 3.2.2.**

| **CCBs vs other antihypertensive drugs** | | | | | | |
| --- | --- | --- | --- | --- | --- | --- |
| **Patient or population:** patients with hypertension  **Settings:** patients  **Intervention:** CCBs  **Comparison:** other antihypertensive drugs | | | | | | |
| **Outcomes** | **Illustrative comparative risks* (95% CI)** | | **Relative effect (95% CI)** | **No of Participants (studies)** | **Quality of the evidence (GRADE)** | **Comments** |
|  | Assumed risk | Corresponding risk |  |  |  |  |
|  | **other antihypertensive drugs** | **CCBs** |  |  |  |  |
| **CCB VS placebo** | **Study population** | | **OR 0.68**  (0.61 to 0.75) | 43534 (10 studies) | ⊕⊕⊕⊕ **high**^1^ |  |
|  | **42 per 1000** | **29 per 1000** (26 to 32) |  |  |  |  |
|  | **Medium risk population** | |  |  |  |  |
|  | **39 per 1000** | **27 per 1000** (24 to 30) |  |  |  |  |
| **CCB VS β-blockers or diuretics** | **Study population** | | **RR 0.87**  (0.84 to 0.9) | 397880 (16 studies) | ⊕⊕⊕⊕ **high**^1^ |  |
|  | **35 per 1000** | **30 per 1000** (29 to 31) |  |  |  |  |
|  | **Medium risk population** | |  |  |  |  |
|  | **26 per 1000** | **23 per 1000** (22 to 23) |  |  |  |  |
| **CCBs vs β-blockers and Diuretics** | **Study population** | | **OR 0.89**  (0.83 to 0.95) | 108044 (6 studies) | ⊕⊕⊕⊕ **high**^1^ |  |
|  | **35 per 1000** | **31 per 1000** (29 to 33) |  |  |  |  |
|  | **Medium risk population** | |  |  |  |  |
|  | **31 per 1000** | **28 per 1000** (26 to 29) |  |  |  |  |
| **CCBs vs Diuretics** | **Study population** | | **OR 0.95**  (0.84 to 1.07) | 28199 (6 studies) | ⊕⊕⊕⊕ **high**^1^ |  |
|  | **42 per 1000** | **40 per 1000** (36 to 45) |  |  |  |  |
|  | **Medium risk population** | |  |  |  |  |
|  | **16 per 1000** | **15 per 1000** (13 to 17) |  |  |  |  |
| **CCBs vs β-blockers** | **Study population** | | **OR 0.79**  (0.72 to 0.87) | 62697 (4 studies) | ⊕⊕⊕⊕ **high**^1^ |  |
|  | **32 per 1000** | **25 per 1000** (23 to 28) |  |  |  |  |
|  | **Medium risk population** | |  |  |  |  |
|  | **28 per 1000** | **22 per 1000** (20 to 24) |  |  |  |  |
| **CCB VS ACEI** | **Study population** | | **OR 0.92**  (0.83 to 1.02) | 31069 (8 studies) | ⊕⊕⊕⊕ **high**^1^ |  |
|  | **51 per 1000** | **47 per 1000** (43 to 52) |  |  |  |  |
|  | **Medium risk population** | |  |  |  |  |
|  | **38 per 1000** | **35 per 1000** (32 to 39) |  |  |  |  |
| *The basis for the **assumed risk** (e.g. the median control group risk across studies) is provided in footnotes. The **corresponding risk** (and its 95% confidence interval) is based on the assumed risk in the comparison group and the **relative effect** of the intervention (and its 95% CI).  **CI:** Confidence interval; **RR:** Risk ratio; **OR:** Odds ratio; | | | | | | |
| GRADE Working Group grades of evidence  **High quality:** Further research is very unlikely to change our confidence in the estimate of effect.  **Moderate quality:** Further research is likely to have an important impact on our confidence in the estimate of effect and may change the estimate.  **Low quality:** Further research is very likely to have an important impact on our confidence in the estimate of effect and is likely to change the estimate.  **Very low quality:** We are very uncertain about the estimate. | | | | | | |
| ^1^ We only collected published randomized controlled trials. | | | | | | |

**CCBs vs other antihypertensive drugs**

| **Quality assessment** | | | | | | | **Summary of findings** | | | | | **Importance** |
| --- | --- | --- | --- | --- | --- | --- | --- | --- | --- | --- | --- | --- |
|  |  |  |  |  |  |  | **No of patients** | | **Effect** | | **Quality** |  |
| **No of studies** | **Design** | **Limitations** | **Inconsistency** | **Indirectness** | **Imprecision** | **Other considerations** | **CCBs** | **other antihypertensive drugs** | **Relative (95% CI)** | **Absolute** |  |  |
| **CCB VS placebo** | | | | | | | | | | | | |
| 10 | randomised trials | no serious limitations | no serious inconsistency | no serious indirectness | no serious imprecision | none | 622/21844 (2.8%) | 907/21690 (4.2%) | OR 0.68 (0.61 to 0.75) | 13 fewer per 1000 (from 10 fewer to 16 fewer) | ⊕⊕⊕⊕ HIΓH | IMPORTANT |
|  |  |  |  |  |  |  |  | 3.9% |  | 12 fewer per 1000 (from 9 fewer to 15 fewer) |  |  |
| **CCB VS β-blockers or diuretics** | | | | | | | | | | | | |
| 16 | randomised trials | no serious limitations | no serious inconsistency | no serious indirectness | no serious imprecision | none | 5590/186222 (3%) | 7420/211658 (3.5%) | RR 0.87 (0.84 to 0.9) | 5 fewer per 1000 (from 4 fewer to 6 fewer) | ⊕⊕⊕⊕ HIΓH | IMPORTANT |
|  |  |  |  |  |  |  |  | 2.6% |  | 3 fewer per 1000 (from 3 fewer to 4 fewer) |  |  |
| **CCBs vs β-blockers and Diuretics** | | | | | | | | | | | | |
| 6 | randomised trials | no serious limitations | no serious inconsistency | no serious indirectness | no serious imprecision | none | 1580/50725 (3.1%) | 2003/57319 (3.5%) | OR 0.89 (0.83 to 0.95) | 4 fewer per 1000 (from 2 fewer to 6 fewer) | ⊕⊕⊕⊕ HIΓH | IMPORTANT |
|  |  |  |  |  |  |  |  | 3.1% |  | 3 fewer per 1000 (from 2 fewer to 5 fewer) |  |  |
| **CCBs vs Diuretics** | | | | | | | | | | | | |
| 6 | randomised trials | no serious limitations | no serious inconsistency | no serious indirectness | no serious imprecision | none | 424/11001 (3.9%) | 720/17198 (4.2%) | OR 0.95 (0.84 to 1.07) | 2 fewer per 1000 (from 6 fewer to 3 more) | ⊕⊕⊕⊕ HIΓH | IMPORTANT |
|  |  |  |  |  |  |  |  | 1.6% |  | 1 fewer per 1000 (from 3 fewer to 1 more) |  |  |
| **CCBs vs β-blockers** | | | | | | | | | | | | |
| 4 | randomised trials | no serious limitations | no serious inconsistency | no serious indirectness | no serious imprecision | none | 791/31385 (2.5%) | 987/31312 (3.2%) | OR 0.79 (0.72 to 0.87) | 6 fewer per 1000 (from 4 fewer to 9 fewer) | ⊕⊕⊕⊕ HIΓH | IMPORTANT |
|  |  |  |  |  |  |  |  | 2.8% |  | 6 fewer per 1000 (from 4 fewer to 8 fewer) |  |  |
| **CCB VS ACEI** | | | | | | | | | | | | |
| 8 | randomised trials | no serious limitations | no serious inconsistency | no serious indirectness | no serious imprecision | none | 728/15511 (4.7%) | 788/15558 (5.1%) | OR 0.92 (0.83 to 1.02) | 4 fewer per 1000 (from 8 fewer to 1 more) | ⊕⊕⊕⊕ HIΓH | IMPORTANT |
|  |  |  |  |  |  |  |  | 3.8% |  | 3 fewer per 1000 (from 6 fewer to 1 more) |  |  |
