# Supplementary material for: The Effects of Calcium Channel Blockers in the Prevention of Stroke in Adults with Hypertension: A Meta-Analysis of Data from 273,543 Participants in 31 Randomized Controlled Trials
Source: PLoS One. 2013 Mar 6;8(3):e57854. doi: 10.1371/journal.pone.0057854 (PMC3590278; doi:10.1371/journal.pone.0057854)
Supplement: Supplementary Information S2 — The risk of bias assessment by RevMan. (DOC) [file pone.0057854.s002.doc]

**Supplementary Information 2**

**The risk of bias assessment for each included study by RevMan version 5.0.**


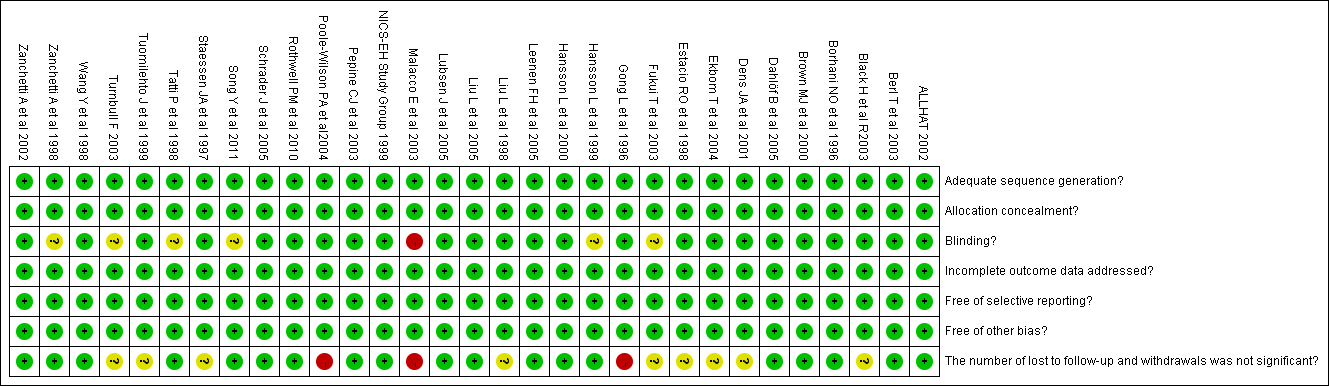


**
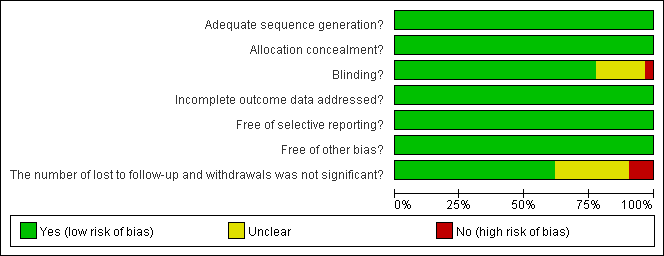
**

**The risk of bias assessment for each included study**

1. **CCBs vs Placebo for reducing the incidence of stroke**

Poole-Wilson PA et al 2004

#### Risk of bias table

| **Item** | **Judgement** | **Description** |
| --- | --- | --- |
| Adequate sequence generation? | Yes | Quote:"Randomization was performed centrally by computer-generated code"  Comment: multi-central randomization |
| Allocation concealment? | Yes | Quote:"Randomization was performed centrally by computer-generated code"  Comment: multi-central randomization |
| Blinding? | Yes | Quote:"double-blind, placebo controlled study"  Comment: definitely yes |
| Incomplete outcome data addressed? | Yes | Quote:"The number of patients that lost to follow-up was carefully described.491 lost in experimental group,470 lost in control group"  Comment: the remaining patients were 100% follow up |
| Free of selective reporting? | Yes | All outcomes listed in methods section are reported on in the results section |
| Free of other bias? | Yes | Study was stopped for insufficient accrual but not for benefit |
| The number of lost to follow-up and withdrawals was not significant? | No | 491 lost in experimental group,470 lost in control group |

Lubsen J et al 2005

#### Risk of bias table

| **Item** | **Judgement** | **Description** |
| --- | --- | --- |
| Adequate sequence generation? | Yes | Quote: The sequence of treatment was randomly assigned in blocks of constant size"  Comment: multi-central , randomization |
| Allocation concealment? | Yes | Quote:"Randomization was performed centrally by computer-generated code"  Comment: multi-central randomization |
| Blinding? | Yes | Quote:"double-blind, placebo controlled study"  Comment: definitely yes |
| Incomplete outcome data addressed? | Yes | All outcomes listed in methods section are reported on in the results section |
| Free of selective reporting? | Yes | Study was stopped for insufficient accrual but not for benefit |
| Free of other bias? | Yes | Study was stopped for insufficient accrual but not for benefit |
| The number of lost to follow-up and withdrawals was not significant? | Yes | The number of patients that lost to follow-up was described carefully due to the specific reason |

Turnbull F 2003

#### Risk of bias table

| **Item** | **Judgement** | **Description** |
| --- | --- | --- |
| Adequate sequence generation? | Yes | Quote" Prospectively-designed overviews with data from 29 randomized trials" |
| Allocation concealment? | Yes | Quote:"Data were checked for completeness and balance between randomized groups, and were reviewed for accuracy on at least two occasions" |
| Blinding? | Unclear | Quote: "prospectively-designed overviews with data from 29 randomized trials"  Comment: probably yes |
| Incomplete outcome data addressed? | Yes | Data were checked for completeness and balance between randomized groups, and were reviewed for accuracy on at least two occasions |
| Free of selective reporting? | Yes | All outcomes of interest reported |
| Free of other bias? | Yes | Study was stopped for insufficient accrual but not for benefit |
| The number of lost to follow-up and withdrawals was not significant? | Unclear | The number of patients that lost to follow-up was described carefully due to the specific reason. However study does not report number of lost to follow-up events |

Liu L et al 2005

#### Risk of bias table

| **Item** | **Judgement** | **Description** |
| --- | --- | --- |
| Adequate sequence generation? | Yes | Quote: "Study was an investigator-designed, prospective, multi-centre, double-bind, randomized, placebo-controlled, parallel-group trial" |
| Allocation concealment? | Yes | Quote:"It was supported by the Chinese Ministry of Public Health and the Ministry of Science, as a project of the Ninth National Five-Year Plan" |
| Blinding? | Yes | Quote:"an investigator-designed, prospective, multicentre, double-blind, randomized, placebo-controlled, parallel group trial"  Comment: definitely yes |
| Incomplete outcome data addressed? | Yes | Quote:"Most of patients were observed until the end of the study "."14 lost in experimental group,16 lost in control group" |
| Free of selective reporting? | Yes | All outcomes listed in methods section are reported on in the results section |
| Free of other bias? | Yes | All outcomes of interest reported |
| The number of lost to follow-up and withdrawals was not significant? | Yes | The number of patients that lost to follow-up was described carefully due to the specific reason |

Berl T et al 2003

#### Risk of bias table

| **Item** | **Judgement** | **Description** |
| --- | --- | --- |
| Adequate sequence generation? | Yes | Quote:"The sequence of treatments was randomized double-blind, placebo-controlled designed"  Comment: definitely yes |
| Allocation concealment? | Yes | Quote:"Patients were randomly assigned centrally by computer to receive treatment"  Comment: definitely yes |
| Blinding? | Yes | The study was a randomized, double-blind study on the effect of treatment. The protocol of this study has been published |
| Incomplete outcome data addressed? | Yes | Quote:"No patients was lost to follow-up"  Comment:100% follow up |
| Free of selective reporting? | Yes | The protocol of this study has been published. Setting: 209 centers in the Americas, Europe, Israel, and Australasia. The institutional review boards of each center approved the protocol |
| Free of other bias? | Yes | All outcomes of interest reported |
| The number of lost to follow-up and withdrawals was not significant? | Yes | No patients was lost to follow-up |

Tuomilehto J et al 1999

#### Risk of bias table

| **Item** | **Judgement** | **Description** |
| --- | --- | --- |
| Adequate sequence generation? | Yes | Quote:"The patients were randomly assigned to double-blind treatment with active medication or placebo by means of a computer-generated schedule"  Comment: definitely yes |
| Allocation concealment? | Yes | Quote:"The protocol of the trial was approved by the ethics committees of the University of Leuven, Leuven, Belgium, and the participating centers and was implemented according to the Declaration of Helsinki" |
| Blinding? | Yes | The patients were randomly assigned to double-blind treatment with active medication or placebo |
| Incomplete outcome data addressed? | Yes | All outcomes listed in methods section are reported on in the results section |
| Free of selective reporting? | Yes | All outcomes of interest reported |
| Free of other bias? | Yes | Study was stopped for insufficient accrual but not for benefit |
| The number of lost to follow-up and withdrawals was not significant? | Unclear | The number of patients that lost to follow-up was described carefully due to the specific reason. However study does not report number of lost to follow-up events |

Dens JA et al 2001

#### Risk of bias table

| **Item** | **Judgement** | **Description** |
| --- | --- | --- |
| Adequate sequence generation? | Yes | Quote:"This was a randomized, double-blind, single-center study approved by the Ethical Committee of our institution" |
| Allocation concealment? | Yes | Quote:"This was a randomized, double-blind, single-center study approved by the Ethical Committee of our institution"  Comment: central randomization |
| Blinding? | Yes | This was a randomized, double-blind, single-center study |
| Incomplete outcome data addressed? | Yes | Convincing data will only be available at the end of the 3-year follow-up period |
| Free of selective reporting? | Yes | All outcomes of interest reported |
| Free of other bias? | Yes | No reported |
| The number of lost to follow-up and withdrawals was not significant? | Unclear | The number of patients that lost to follow-up was described carefully due to the specific reason. However study does not report number of lost to follow-up events |

Gong L et al 1996

#### Risk of bias table

| **Item** | **Judgement** | **Description** |
| --- | --- | --- |
| Adequate sequence generation? | Yes | Quote:"A single-blind trial was conducted under the direction of the Shanghai Institute of Hypertension |
| Allocation concealment? | Yes | Quote:"Clinical events and risk modification were analyzed in collaboration with the university of Montreal" |
| Blinding? | Yes | The study was performed as a single-blind, placebo-controlled prospective trial |
| Incomplete outcome data addressed? | Yes | Quote:"The number of patients that lost to follow-up was carefully described. 134 lost in experimental group,174 lost in control group"  Comment: the remaining patients were 100% follow up |
| Free of selective reporting? | Yes | All outcomes of interest reported |
| Free of other bias? | Yes | Study not reported |
| The number of lost to follow-up and withdrawals was not significant? | No | 134 lost in experimental group,174 lost in control group |

Liu L et al 1998

#### Risk of bias table

| **Item** | **Judgement** | **Description** |
| --- | --- | --- |
| Adequate sequence generation? | Yes | Quote:"A single-blind trial was conducted under the direction of the World Health Organization and the World Hypertension League. |
| Allocation concealment? | Yes | Quote:"The trial was carried out in consultation with the World Health Organization and the World Hypertension League" |
| Blinding? | Yes | Quote:"single-blind, placebo controlled study"  Comment: definitely yes |
| Incomplete outcome data addressed? | Yes | All outcomes listed in methods section are reported on in the results section.115 lost in experimental group,122 lost in control group |
| Free of selective reporting? | Yes | Study not reported |
| Free of other bias? | Yes | All outcomes listed in methods section are reported on in the results section |
| The number of lost to follow-up and withdrawals was not significant? | Unclear | 115 lost in experimental group,122 lost in control group |

Staessen JA et al 1997

#### Risk of bias table

| **Item** | **Judgement** | **Description** |
| --- | --- | --- |
| Adequate sequence generation? | Yes | Quote:"Randomization was performed by the intention-to-treat analysis" |
| Allocation concealment? | Yes | Quote:"The protocol of this trial was approved by the ethics committees of the University of Leuven and the participating centre ,this trial used the principles outlined in the Helsinki declaration" |
| Blinding? | Yes | Quote:"double-blind, placebo controlled study"  Comment: definitely yes |
| Incomplete outcome data addressed? | Yes | Patients without any report within the year before the trial stopped were counted as lost to follow-up |
| Free of selective reporting? | Yes | All outcomes listed in methods section are reported on in the results section |
| Free of other bias? | Yes | Study not reported |
| The number of lost to follow-up and withdrawals was not significant? | Unclear | 121 lost in experimental group,116 lost in control group |

1. **CCBs vs ACEIs for reduce the incidence of stroke**

Estacio RO et al 1998

#### Risk of bias table

| **Item** | **Judgement** | **Description** |
| --- | --- | --- |
| Adequate sequence generation? | Yes | Quote:"Randomization was previously designed" |
| Allocation concealment? | Yes | Quote:"The design of the trial has been described previously. The study was approved by the institutional review board of the University of Colorado Health Sciences Center. All patients gave written informed consent" |
| Blinding? | Yes | Quote:"prospective, randomized, blinded study"  Comment: definitely yes |
| Incomplete outcome data addressed? | Yes | All outcomes listed in methods section are reported on in the results section |
| Free of selective reporting? | Yes | Quote:"Since the findings were based on a secondary end point of the study, the results should be interpreted cautiously"  Comment: probably yes |
| Free of other bias? | Yes | No reported |
| The number of lost to follow-up and withdrawals was not significant? | Unclear | The number of patients that lost to follow-up was described carefully due to the specific reason. However study does not report number of lost to follow-up events |

Leenen FH et al 2005

#### Risk of bias table

| **Item** | **Judgement** | **Description** |
| --- | --- | --- |
| Adequate sequence generation? | Yes | Quote:"Data were analyzed according to participants' randomized treatment assignments (intent-to-treat analysis)"  Comment: definitely yes |
| Allocation concealment? | Yes | Quote:"The National Heart, Lung, and Blood Institute sponsored the study and involved in all aspects other than direct operations of the study centers. This included collection, analysis, and interpretation of the data plus the decision to submit the article for publication" |
| Blinding? | Yes | A randomized, double-blind, multicenter clinical trial sponsored by the National Heart, Lung, and Blood Institute |
| Incomplete outcome data addressed? | Yes | All outcomes listed in methods section are reported on in the results section.258 lost in experimental group,276 lost in control group |
| Free of selective reporting? | Yes | All outcomes of interest reported |
| Free of other bias? | Yes | The distribution of baseline factors was similar for the 2 groups |
| The number of lost to follow-up and withdrawals was not significant? | Yes | At trial closeout, 258 (2.8%) of the amlodipine group and 276 (3.0%) of the lisinopril group had unknown vital status |

Fukui T et al 2003

#### Risk of bias table

| **Item** | **Judgement** | **Description** |
| --- | --- | --- |
| Adequate sequence generation? | Yes | Quote:"This trial was a prospective, multicenter, randomized, open-label, active-controlled, 2-arm parallel group" |
| Allocation concealment? | Yes | Quote:"Data management will be conducted at the EBM Collaborative Research Center of the Kyoto University Graduate School of Medicine using the Automatic Bar Code Data-Capturing/Allocation, Booking &Trail Coding, Data Management TM (ABCDTM) system, a novel Internet system for data collection and management" |
| Blinding? | Unclear | Quote:"This trial was a prospective, multicenter, randomized, open-label, active-controlled, 2-arm parallel group"  Comment: probably yes |
| Incomplete outcome data addressed? | Yes | All outcomes listed in methods section are reported on in the results section |
| Free of selective reporting? | Yes | All outcomes of interest reported |
| Free of other bias? | Yes | No reported |
| The number of lost to follow-up and withdrawals was not significant? | Unclear | The number of patients that lost to follow-up was described carefully due to the specific reason. However study does not report number of lost to follow-up events |

Song Y et al 2011

#### Risk of bias table

| **Item** | **Judgement** | **Description** |
| --- | --- | --- |
| Adequate sequence generation? | Yes | Quote:"Randomization was performed centrally by computer-generated code" |
| Allocation concealment? | Yes | Quote:"Randomization was performed centrally by computer-generated code"  Comment: central randomization |
| Blinding? | Unclear | Quote:"double-blind, placebo controlled study"  Comment: probably no |
| Incomplete outcome data addressed? | Yes | All outcomes listed in methods section are reported on in the results section |
| Free of selective reporting? | Yes | All outcomes of interest reported |
| Free of other bias? | Yes | No reported |
| The number of lost to follow-up and withdrawals was not significant? | Yes | No patient was lost to follow-up and withdrew |

Tatti P et al 1998

#### Risk of bias table

| **Item** | **Judgement** | **Description** |
| --- | --- | --- |
| Adequate sequence generation? | Yes | Quote:"Randomization was performed centrally by computer-generated code" |
| Allocation concealment? | Yes | Quote:"Randomization was performed centrally by computer-generated code"  Comment: central randomization |
| Blinding? | Unclear | Quote:"Randomization was designed as an open-label, randomized prospective trial" |
| Incomplete outcome data addressed? | Yes | All outcomes listed in methods section are reported on in the results section |
| Free of selective reporting? | Yes | All outcomes of interest reported |
| Free of other bias? | Yes | Study not reported |
| The number of lost to follow-up and withdrawals was not significant? | Yes | 3 lost in experimental group,1 lost in control group |

Hansson L et al 1999

#### Risk of bias table

| **Item** | **Judgement** | **Description** |
| --- | --- | --- |
| Adequate sequence generation? | Yes | Quote:"a prospective, randomized , open, masked-endpoint trial was designed" |
| Allocation concealment? | Yes | Quote:"a prospective, randomized , open, masked-endpoint trial was designed, which is similar to routine clinical practice"  Comment: definitely yes |
| Blinding? | Unclear | Quote:"double-blind, placebo controlled study"  Comment: probably no |
| Incomplete outcome data addressed? | Yes | All outcomes of interest reported |
| Free of selective reporting? | Yes | All outcomes listed in methods section are reported on in the results section |
| Free of other bias? | Yes | Study not reported |
| The number of lost to follow-up and withdrawals was not significant? | Yes | No patient was lost to follow-up and no patient refused to continue in the study |

Schrader J et al 2005

#### Risk of bias table

| **Item** | **Judgement** | **Description** |
| --- | --- | --- |
| Adequate sequence generation? | Yes | Quote:"Randomization was successful without significant differences in the baseline characteristics. All results was based on intention to treatment analyses" |
| Allocation concealment? | Yes | Quote:"This was an investigator-initiated study involving patients from internal medicine and general medicine practices and hospitals in Germany and Austria" |
| Blinding? | Yes | This was a prospective, randomized, controlled and multicenter trial. A blinded end point committee assessed all cerebrovascular and cardiovascular events |
| Incomplete outcome data addressed? | Yes | All outcomes listed in methods section are reported on in the results section |
| Free of selective reporting? | Yes | All outcomes of interest reported |
| Free of other bias? | Yes | Study not reported |
| The number of lost to follow-up and withdrawals was not significant? | Yes | 12 lost in experimental group,14 lost in control group |

Ekbom T et al 2004

#### Risk of bias table

| **Item** | **Judgement** | **Description** |
| --- | --- | --- |
| Adequate sequence generation? | Yes | Quote:"Analysis was by intention to treat" |
| Allocation concealment? | Yes | Quote:"Consenting patient was endorsed by the Swedish Society of Hypertension"  Comment: definitely yes |
| Blinding? | Yes | Quote:"double-bind, placebo controlled study"  Comment: probably yes |
| Incomplete outcome data addressed? | Yes | Although not clearly stated,100% of patient randomized were analyzed  Comment: probably yes |
| Free of selective reporting? | Yes | All outcomes of interest reported |
| Free of other bias? | Yes | The patient population and the study design have been described previously. There were no relevant differences in respect to the history of cardiovascular disease between the different treatment groups |
| The number of lost to follow-up and withdrawals was not significant? | Unclear | The number of patients that lost to follow-up was described carefully due to the specific reason. However study does not report number of lost to follow-up events |

1. **CCBs compared to β blockers or/and Diuretics for hypertension**

ALLHAT 2002

#### Risk of bias table

| **Item** | **Judgement** | **Description** |
| --- | --- | --- |
| Adequate sequence generation? | Yes | Quote:"Randomization handled through the National Heart, Lung, and Blood Institute" |
| Allocation concealment? | Yes | Quote:"Randomization handled through the National Heart, Lung, and Blood Institute"  Comment: central randomization |
| Blinding? | Yes | Quote:"Randomized, double-blind, multicenter clinical trial"  Comment: definitely yes |
| Incomplete outcome data addressed? | Yes | All outcomes listed in methods section are reported on in the results section |
| Free of selective reporting? | Yes | All outcomes of interest reported |
| Free of other bias? | Yes | No reported |
| The number of lost to follow-up and withdrawals was not significant? | Yes | 258 lost in experimental group,419 lost in control group |

Rothwell PM et al 2010

#### Risk of bias table

| **Item** | **Judgement** | **Description** |
| --- | --- | --- |
| Adequate sequence generation? | Yes | Quote:"Randomization was done on an intention-to-treat basis" |
| Allocation concealment? | Yes | Quote:"Randomization was done on an intention-to-treat basis" |
| Blinding? | Yes | Quote:"Open, prospective randomized, blinded endpoint study"  Comment: definitely yes |
| Incomplete outcome data addressed? | Yes | There were no differences in the number of missing follow-up visits (3.7% overall), and so any bias in estimation of visit-to-visit variability is unlikely. |
| Free of selective reporting? | Yes | Main analyses were done on an intention-to-treat basis |
| Free of other bias? | Yes | Main analyses were done on an intention-to-treat basis. All outcomes of interest reported |
| The number of lost to follow-up and withdrawals was not significant? | Yes | There were no differences in the number of missing follow-up visits (3.7% overall), and so any bias in estimation of visit-to-visit variability is unlikely |

Dahlöf B et al 2005

#### Risk of bias table

| **Item** | **Judgement** | **Description** |
| --- | --- | --- |
| Adequate sequence generation? | Yes | Quote:"The randomization was a computer generated optimum allocation blinded for any person involved in the undertaking of the study" |
| Allocation concealment? | Yes | Quote:"The study conformed to good clinical practice guidelines and was done in accord with the Declaration of Helsinki" |
| Blinding? | Yes | Quote:"an independent, investigator initiated , investigator-led, multicentre, prospective, randomized controlled trial"  Comment: definitely yes |
| Incomplete outcome data addressed? | Yes | Patients gave written informed consent to participate in the trial before randomization. All outcomes listed in methods section are reported on in the results section |
| Free of selective reporting? | Yes | All outcomes listed in methods section are reported on in the results section |
| Free of other bias? | Yes | Quote:"The protocol and all subsequent amendments to the protocol were reviewed and ratified by central and regional ethics review boards in the UK, and by national ethics and statutory bodies in Ireland and the Nordic (Sweden, Denmark, Iceland, Norway, and Finland) countries" |
| The number of lost to follow-up and withdrawals was not significant? | Yes | 121 lost in experimental group,171 lost in control group |

Turnbull F 2003

#### Risk of bias table

| **Item** | **Judgement** | **Description** |
| --- | --- | --- |
| Adequate sequence generation? | Yes | Quote:"Prospectively-designed overviews with data from 29 randomized trials" |
| Allocation concealment? | Yes | Quote:"Data were checked for completeness and balance between randomized groups, and were reviewed for accuracy on at least two occasions" |
| Blinding? | Unclear | Quote:"prospectively-designed overviews with data from 29 randomized trials"  Comment: probably yes |
| Incomplete outcome data addressed? | Yes | Data were checked for completeness and balance between randomized groups, and were reviewed for accuracy on at least two occasions |
| Free of selective reporting? | Yes | All outcomes of interest reported |
| Free of other bias? | Yes | Study was stopped for insufficient accrual but not for benefit |
| The number of lost to follow-up and withdrawals was not significant? | Unclear | The number of patients that lost to follow-up was described carefully due to the specific reason. However study does not report number of lost to follow-up events |

Black H et al R2003

#### Risk of bias table

| **Item** | **Judgement** | **Description** |
| --- | --- | --- |
| Adequate sequence generation? | Yes | Quote:"Participants were randomized from 661 clinical sites in 15 countries. All had signed informed consent" |
| Allocation concealment? | Yes | Quote:"This was a randomized, double-blind, active-controlled, multicenter, international clinical trial" |
| Blinding? | Yes | Quote:"Double-blind, randomized, active-controlled, multicenter clinical study"  Comment: definitely yes |
| Incomplete outcome data addressed? | Yes | After a mean of 3 years of follow-up, the sponsor closed the study before unblinding the results. All outcomes listed in methods section are reported on in the results section |
| Free of selective reporting? | Yes | All study medication for a participant was obtained by using the interactive voice response system |
| Free of other bias? | Yes | All outcomes of interest reported |
| The number of lost to follow-up and withdrawals was not significant? | Unclear | 570 lost in experimental group,563 lost in control group |

Hansson L et al 1999

#### Risk of bias table

| **Item** | **Judgement** | **Description** |
| --- | --- | --- |
| Adequate sequence generation? | Yes | Quote:"a prospective, randomized, open, masked-endpoint trial was designed" |
| Allocation concealment? | Yes | Quote:"a prospective, randomized, open, masked-endpoint trial was designed, which is similar to routine clinical practice"  Comment: definitely yes |
| Blinding? | Unclear | Quote:"double-blind, placebo controlled study"  Comment: probably no |
| Incomplete outcome data addressed? | Yes | All outcomes of interest reported |
| Free of selective reporting? | Yes | All outcomes listed in methods section are reported on in the results section |
| Free of other bias? | Yes | Study not reported |
| The number of lost to follow-up and withdrawals was not significant? | Yes | No patient was lost to follow-up and no patient refused to continue in the study |

Brown MJ et al 2000

#### Risk of bias table

| **Item** | **Judgement** | **Description** |
| --- | --- | --- |
| Adequate sequence generation? | Yes | Quote:"This study was designed to have a statistical power of 90% for an intention-to-treat analysis to detect a 25% relative difference in the primary outcome in a two-sided test at 5% significance between nifedipine and co-amilozide" |
| Allocation concealment? | Yes | Quote:"This study used dynamic randomization to prospectively assign almost equal numbers of patients to the two groups for the common risk factors" |
| Blinding? | Yes | Quote:"Double-blind prospective, randomized, double-blind study"  Comment: definitely yes |
| Incomplete outcome data addressed? | Yes | Patients gave written informed consent to participate in the trial before randomization. All outcomes listed in methods section are reported on in the results section |
| Free of selective reporting? | Yes | All outcomes listed in methods section are reported on in the results section |
| Free of other bias? | Yes | All outcomes of interest reported |
| The number of lost to follow-up and withdrawals was not significant? | Yes | 66 lost in experimental group,83 lost in control group |

Pepine CJ et al 2003

#### Risk of bias table

| **Item** | **Judgement** | **Description** |
| --- | --- | --- |
| Adequate sequence generation? | Yes | Quote:"Study was an international, multicenter study with a prospective, randomized, open blinded end-point evaluation design conducted according to principles of the Declaration of Helsinki" |
| Allocation concealment? | Yes | Quote:"The institutional review boards and ethics committees at participating sites approved the protocol and patients provided written informed consent"  Comment: definitely yes |
| Blinding? | Yes | Quote:"Randomized, prospective, open label, blinded end point study"  Comment: definitely yes |
| Incomplete outcome data addressed? | Yes | Quote:"The number of patients that lost to follow-up was carefully described. 300 lost in experimental group,268 lost in control group"  Comment: the remaining patients were 100% follow up |
| Free of selective reporting? | Yes | All outcomes listed in methods section are reported on in the results section |
| Free of other bias? | Yes | Study was stopped for insufficient accrual but not for benefit |
| The number of lost to follow-up and withdrawals was not significant? | Yes | Patient follow-up was complete when a final assessment form was received via the online data system or a death report was received. For all patients not completing the final assessment visit, lost to follow-up, or withdrawn, data were censored according to last visit date. |

Borhani NO et al 1996

#### Risk of bias table

| **Item** | **Judgement** | **Description** |
| --- | --- | --- |
| Adequate sequence generation? | Yes | Quote:"All case-report forms were examined for evidence of any clinical events and adverse reactions. All reported clinical events were reviewed, adjudicated, and classified by the MIDAS Investigators' Morbidity and Mortality Committee" |
| Allocation concealment? | Yes | Quote:"All case-report forms were examined for evidence of any clinical events and adverse reactions. All reported clinical events were reviewed, adjudicated, and classified by the MIDAS Investigators' Morbidity and Mortality Committee" |
| Blinding? | Yes | Quote:"Randomized, double-blind, multicenter, positive-controlled trial"  Comment :definitely yes |
| Incomplete outcome data addressed? | Yes | Quote:"No patients was lost to follow-up"  Comment:100% follow up |
| Free of selective reporting? | Yes | All outcomes of interest reported |
| Free of other bias? | Yes | No reported |
| The number of lost to follow-up and withdrawals was not significant? | Yes | No patient was lost to follow-up and withdrew |

Wang Y et al 1998

#### Risk of bias table

| **Item** | **Judgement** | **Description** |
| --- | --- | --- |
| Adequate sequence generation? | Yes | Quote:"The randomization list was generated by an independent statistician" |
| Allocation concealment? | Yes | Quote:"The randomization list was generated by an independent statistician" |
| Blinding? | Yes | Quote:"single-blind, placebo controlled study"  Comment: probably yes |
| Incomplete outcome data addressed? | Yes | Quote:"No patients was lost to follow-up"  Comment:100% follow up |
| Free of selective reporting? | Yes | All outcomes of interest reported |
| Free of other bias? | Yes | No reported |
| The number of lost to follow-up and withdrawals was not significant? | Yes | No patient was lost to follow-up and withdrew |

Hansson L et al 2000

#### Risk of bias table

| **Item** | **Judgement** | **Description** |
| --- | --- | --- |
| Adequate sequence generation? | Yes | Quote:"Analysis was done by intention to treat" |
| Allocation concealment? | Yes | Quote:"Investigators called the randomization centre at Clinical Data Care in Lund, Sweden, to obtain randomization numbers and treatment assignment" |
| Blinding? | Yes | Quote:"prospective, randomized, open, blinded endpoint study"  Comment: definitely yes |
| Incomplete outcome data addressed? | Yes | All outcomes listed in methods section are reported on in the results section |
| Free of selective reporting? | Yes | All endpoints were assessed by an independent endpoint committee, according to strict and prespecified criteria for the approval of endpoints |
| Free of other bias? | Yes | All outcomes of interest reported |
| The number of lost to follow-up and withdrawals was not significant? | Yes | 24 lost in experimental group,28 lost in control group |

NICS-EH Study Group 1999

#### Risk of bias table

| **Item** | **Judgement** | **Description** |
| --- | --- | --- |
| Adequate sequence generation? | Yes | Quote:"The details of the design of and methods used in the study have been published previously" |
| Allocation concealment? | Yes | Quote:"For patients who had any end point, the attending physician’s judgment was assessed blindly by the Steering Committee and the diagnosis was confirmed" |
| Blinding? | Yes | Quote:"double-blind, placebo controlled study"  Comment: definitely yes |
| Incomplete outcome data addressed? | Yes | Quote:"The number of patients that lost to follow-up was carefully described. 6 lost in experimental group, 9 lost in control group"  Comment: the remaining patients were 100% follow up |
| Free of selective reporting? | Yes | All outcomes listed in methods section are reported on in the results section |
| Free of other bias? | Yes | Study not reported |
| The number of lost to follow-up and withdrawals was not significant? | Yes | 6 lost in experimental group, 9 lost in control group |

Malacco E et al 2003

#### Risk of bias table

| **Item** | **Judgement** | **Description** |
| --- | --- | --- |
| Adequate sequence generation? | Yes | Quote:"Data were analyzed on an intention-to-treat basis by BETA Trial Center." |
| Allocation concealment? | Yes | Quote:"The trial had a multicenter structure and the centers were selected mainly among geriatric and internal medicine centers in charge of elderly outpatients in connections with family doctors to make it as representative as possible of the clinical practice" |
| Blinding? | No | Quote:"Open, prospective, randomized designed study"  Comment: probably yes |
| Incomplete outcome data addressed? | Yes | Patients lost to follow-up were 12.3% in the lacidipine and 11% in the chlorthalidone group, respectively |
| Free of selective reporting? | Yes | All outcomes listed in methods section are reported on in the results section |
| Free of other bias? | Yes | The overall incidence of the primary endpoints was 9.3% with no signiﬁcant between group difference |
| The number of lost to follow-up and withdrawals was not significant? | No | 116 lost in experimental group, 104 lost in control group |

Zanchetti A et al 2002

#### Risk of bias table

| **Item** | **Judgement** | **Description** |
| --- | --- | --- |
| Adequate sequence generation? | Yes | Quote:"Randomization was performed by the intention-to-treat analysis" |
| Allocation concealment? | Yes | Quote:"Events were examined 3 times during the study by an Independent Safety Committee in an unblinded manner" |
| Blinding? | Yes | Quote:"double-blind, placebo controlled study"  Comment: definitely yes |
| Incomplete outcome data addressed? | Yes | This study randomly allocated 2334 patients to double-blind treatment (safety population, with 43 atenolol and 49 lacidipine patients lost to follow-up) |
| Free of selective reporting? | Yes | All outcomes of interest reported |
| Free of other bias? | Yes | No significant difference between treatments was found in any cardiovascular event |
| The number of lost to follow-up and withdrawals was not significant? | Yes | 49 lost in experimental group, 43 lost in control group |

Zanchetti A et al 1998

#### Risk of bias table

| **Item** | **Judgement** | **Description** |
| --- | --- | --- |
| Adequate sequence generation? | Yes | Quote:"All randomized patients were included in the ﬁnal analysis according to intention-to- treat procedures" |
| Allocation concealment? | Yes | Quote:"All statistical analyses were done by an independent statistical center. BMDP statistical software programs (BMDP Software, Los Angeles, California, USA) were used" |
| Blinding? | Unclear | Quote:"Prospective, multicenter, randomized, parallel-group, clinical study"  Comment: probably yes |
| Incomplete outcome data addressed? | Yes | Quote:"No patients was lost to follow-up"  Comment:100% follow up |
| Free of selective reporting? | Yes | Per protocol analyses limited to the patients remaining on randomized treatment throughout the trial were also performed, for descriptive purposes only |
| Free of other bias? | Yes | All outcomes of interest reported |
| The number of lost to follow-up and withdrawals was not significant? | Yes | No patient was lost to follow-up and withdrew |

The criteria and interpretation for the assessment risk of bias on each important outcome (across domains) within and across studies.

**Low risk of bias:** Plausible bias unlikely to seriously alter the results, low risk of bias for all key domains (within a study), and most information is from studies at low risk of bias (across studies).

**Unclear risk of bias:** That raises some doubt about the results, unclear risk of bias for one or more key domains (within a study), and most information is from studies at low or unclear risk of bias (across studies).

**High risk of bias:** Plausible bias that seriously weakens confidence in the results, high risk of bias for one or more key domains (within a study), the proportion of information from studies at high risk of bias is sufficient to affect the interpretation of results (across studies).
